# Factors affecting attitudes toward migrants—An evolutionary approach

**DOI:** 10.1002/ajhb.23435

**Published:** 2020-05-26

**Authors:** Alexander Schahbasi, Susanne Huber, Martin Fieder

**Affiliations:** ^1^ Department of Evolutionary Anthropology University of Vienna Vienna Austria; ^2^ Erlangen Centre for Islam & Law in Europe Friedrich‐Alexander University Erlangen‐Nürnberg Erlangen Germany

## Abstract

**Objective:**

To understand migration from an evolutionary perspective, this phenomenon has so far been mainly investigated in animal species. We therefore aim to investigate the potential evolutionary roots of attitudes toward migrants in humans.

**Methods:**

We used data from the European Social Survey (n = 83 734), analyzing attitudes toward migrants by performing ordinal mixed models.

**Results:**

We found that men have a more restrictive attitude toward migration than women, which increases with age and is stronger with a child in the household. Attitude toward migrants is also more skeptical if migrants have a different ethnicity and are from poorer countries. Increasing education and religiousness are associated with a more positive attitude toward migrants, particularly toward migrants of different ethnicity and from poorer countries.

**Discussion:**

Although migration flows are a hallmark of the human species, previous findings suggest that (pre‐)historic migration flows were at times accompanied by conflict and violence, while at the same time, they insured survival by allowing cultural exchange and the avoidance of inbreeding. Accordingly, we assume that contemporary attitudes toward migration are rooted in our evolutionary past. We discuss the respective behavioral patterns from an evolutionary perspective, arguing that both—a negative attitude as well as openness—make sense.

## 1 INTRODUCTION

Migration is one of the most widespread phenomena in nature. In fact, understanding migration as an evolutionary process leads to the assumption that migration must have led to selective benefits for those who migrated. The main causes for migration are usually ecological factors, like seasonality, distributions of resources, habitats, predation and competition, and the quest for mating partners. Typically, there is a balance between benefits and costs of migration (Alerstam, Hedenström, & Åkesson, 2003). Thus, albeit the costs that migration inflicts, there are indications for selection through migration in several species (Fleming, 1975). In many species, migration is associated both with a genetic predisposition to migrate and the ability to learn and the behavioral flexibility to choose, for instance, the best conditions to migrate (Berthold, 1999; Berthold, Helbig, Mohr, & Querner, 1992). Accordingly, the transformation of a resident species into a migrating species and vice versa can be very flexible. In some birds, for instance, this can lead to resident populations, partially migrating populations and fully migrating populations within the same species (Berthold, 1999). While the understanding of migration from an evolutionary perspective has been investigated extensively in many animal species, human migration is most often seen in a merely political, economic, demographic, or humanitarian dimension, while the ultimate evolutionary causes of human migration are not being taken into consideration.

Migration is, however, presumably a heritable trait of the human species (Matthews & Butler, 2011). Throughout history, humans have lived in very diverse ecological environments, which involved an ongoing process of adaptation via surviving and thriving within a new habitat, thus representing a fundamental principle of evolutionary biology (Dobzhansky, 1973). Particularly, the findings of Reich (2018) on the basis of genetic data (ancient DNA) confirm that migration and admixture are long‐standing features of *Homo sapiens* and that we are just at the beginning of understanding the population movements that shaped our genome. Although migration is an feature of the human species, migration flows (particularly the genome shaping prehistoric migrations) could be quite different in nature: while the migration of farmers from Anatolia to Europe 8000 years ago was a rather slow process consisting of large families (Fu et al., 2016), the migration of herders from the Russian plains to Europe 5000 years ago was (due to the domestication of the horse and the use of wheels) much faster and male‐dominated and—as evidence suggests—rather violent with often lethal consequences—particularly for local men (Goldberg, Günther, Rosenberg, & Jakobsson, 2017).

In terms of scale, speed, and space, migration to Europe today differs from these prehistoric flows. Albeit contemporary migration flows are—in relation to resident populations—not as large as in our evolutionary past, they are likely to have an impact on the genomic composition in some countries of destination. This amounts to the fact that migrants usually have a higher fertility compared to the resident population—although only in the first and second generation (Dubuc, 2012). Nevertheless, prehistoric migration flows had a much more substantial impact on the genome of Europeans. From a genetic point of view, the impact of the migration of the Yamnaya from the Steppe (Ukraine/Russia) to Europe on the genome of the resident population, for instance, would be comparable if one billion people would have migrated to present day Germany (Krause & Trappe, 2019). What has changed the impact of migration significantly is the higher population density. Compared to the sparsely populated prehistoric Europe, the increase in population (and urbanization) in the last millennia has not only led to a more densely populated space but also increased social complexity (Schahbasi & Fieder, 2018) and thus rate of human interactions.

Furthermore, migration itself has pronounced effects on the published opinion on the subject, changing from overwhelmingly positive to negative during the massive influx of migrants into Europe from 2014 onward (Berry, Garcia‐Blanco, & Moore, 2016), influencing the attitudes of individuals. Strikingly, contemporary migration and the presence of foreigners frequently evoke highly emotional reactions that oscillate between friendly hospitality and hostile xenophobia. Seen from an evolutionary perspective, these emotional response patterns toward migration and migrants may be understood through the positive and negative evolutionary effects of migration: while migration allowed for an avoidance of in‐breeding (Sikora et al., 2017) and cultural exchange (referring to the exchange of practices and techniques that assured survival), it was—at times—also a violent endeavor with potentially lethal outcomes.

These reaction patterns toward strangers, the attitudes toward foreigners, can also be understood as a simple mechanism of in‐group and out‐group cooperation. This behavioral quirk seems to be a characteristic trait of human behavior: when tested under experimental conditions, even random assignments tend to induce positive in‐group and negative out‐group behavior (Tajfel, Billig, Bundy, & Flament, 1971). Furthermore, also artificial simulations show that ethnocentrism and in‐group favoritism emerge: Hammond and Axelrod (2006) demonstrated that ethnocentrism and in‐group favoritism supports high levels of costly within‐group cooperation, particularly in situations when other more complex mechanisms of cooperation are missing.

The different “branches” of evolutionary behavioral sciences have related concepts on the evolution of “in‐group vs. out‐group attitudes” (reviewed in Salter, 2018). Hence, from the perspective of ethology, Eibl‐Eibesfeldt proposed that community identity and solidarity are an original adaptation from family cohesion (Eibl‐Eibesfeldt, 1979). Relying on observations of families and tribes, he concluded that larger scale cohesion of groups evolved in the past due to maternal and tribal ties, assuming that preadaptation may have evolved by selection on the individual level, but had been stabilized on the level of groups. Accordingly, from early life on, infants bond only with a few reference persons and are displaying—across cultures—a fear of strangers. Interestingly, the display of fear does not depend on negative experiences with strangers as fostering trust within groups is generally accompanied by the avoidance of out‐group members. Eibl‐Eibesfeldt argued that a selection for ties among group members is thus an extension of kin selection on the level of groups (Eibl‐Eibesfeldt, 1982). This group selection approach has been frequently criticized—as have all similar group selection approaches. As argued by *Neo Darwinism* (Dawkins, 1989), individuals who reduce their fitness in favor of non‐kin would be outperformed by selfish individuals and therefore such a behavior must remain nonadaptive. Eibl‐Eibesfeldt later adopted these arguments and argued for multilevel selection (Eibl‐Eibesfeldt, 1982): during our evolution in small groups, even if individuals were not genetically related on the level of close kin, the genetic relatedness was higher as if only estimated in terms of close kin networks. Accordingly, the evolution of in‐group mechanisms may be also explained by the concept of kin selection (Hamilton, 1964, 1971). This approach does not necessarily involve group selection (West, El Mouden, & Gardner, 2011) as cooperation within ethnic groups may be seen as a kind of kin selection on a wider basis (Salter, 2001). Taking this further, humans may behave as “superorganisms” (Wilson, 2010; Wilson & Sober, 1989).

Future molecular genomic and selection studies will provide a more complete picture on the selective forces acting during our evolution (Fieder & Huber, 2016a; Schaschl et al., 2015). Furthermore, balancing selection—different alleles at a selected locus are maintained by selection in a population (Charlesworth, 2006; Relethford, 2012) as a possible mechanism has—to our knowledge—been ignored so far. Thus “in‐group vs. out‐group attitudes” maybe the result of balancing selection. Some individuals in a population may have a friendlier attitude and others a more negative attitude toward members of an out‐group. Overall, this spread of attitudes within a population may have brought reproductive benefits to the bearer depending on the circumstances. There is already evidence for the ongoing balancing selection for the related field of political orientation (Fieder & Huber, 2018).

Migration and the interaction with strangers may both be advantageous as well as detrimental, depending on the parameters of the respective migration flow, as well as the individual sociodemographic profiles of those confronted with migration. Accordingly, beneficial as well as detrimental effects may have led to a balancing selection on the phenotype “*attitude toward strangers*”. In this respect, we aim to investigate the association of individual characteristics such as sex, age, education, and childlessness, as well as attitudes such as religiousness and political orientation on the individual attitude toward migration from the perspective of evolutionary anthropology. From an evolutionary perspective, we would predict that men are more critical of migration than women, as particularly men may have faced a higher risk of death and injury and an increased competition for resources as a result of male dominated migration. Young women, on the other hand, could be expected to be less critical toward migrants as female dispersal was common in many populations. Furthermore, we would expect a more positive attitude toward migrants of those with a higher education, as education is also a sign of the availability of resources and thus reduced competition. Concerning religiousness, our expectations are less clear: On the one hand, we may expect a more open attitude toward strangers due to the integrative power of religions, but on the other hand, we could also expect a more negative attitude because of the rather inclusive character of religions. Expectations with regard to political attitude, however, are very clear as an attitude associated with the political right results in a more negative view of out‐group individuals (Altemeyer, 1998).

To our knowledge, only the European social survey (ESS) uniquely enables to test this association depending on the kind of “out‐group” characteristics of the migrants, differentiating among (a) migration of the same ethnicity, (b) migration of different ethnicity, and (c) of migration from poorer countries.

## 2 METHODS

We used the ESS (https://www.europeansocialsurvey.org/about/) for our analysis. The ESS is an academically driven cross‐national survey that has been conducted across Europe since its establishment in 2001. Every 2 years, face‐to‐face interviews are conducted with newly selected, cross‐sectional samples. The survey measures the attitudes, beliefs, and behavior patterns of the population in more than 30 nations. We pooled the data from all biannual rounds of the ESS from 2002 to 2016 (total eight rounds). We analyzed the data of all participating individuals aged between 18 and 60 years, who had together with their fathers and mothers been born in the surveyed country (totaling 44 223 men and 39 511 women from 33 countries), including only persons who are not member of a group discriminated against (surveyed in the ESS). Note that the sample is biased toward men, and the total number of cases differs substantially among countries (Table 1).

**TABLE 1:** Number of cases for each country

Country	Men	% in Country	Women	% in Country	Total
Austria	465	52.5	421	47.5	886
Belgium	903	53.0	802	47.0	1705
Bulgaria	215	49.4	220	50.6	435
Switzerland	585	53.2	514	46.8	1099
Cyprus	34	54.8	28	45.2	62
Czechia	888	55.4	716	44.6	1604
Germany	8346	55.1	6795	44.9	15 141
Denmark	487	53.7	420	46.3	907
Estonia	69	53.1	61	46.9	130
Spain	4208	55.2	3421	44.8	7629
Finland	601	53.8	517	46.2	1118
France	4069	50.7	3962	49.3	8031
United Kingdom	4094	51.8	3813	48.2	7907
Greece	385	52.1	354	47.9	739
Croatia	58	46.4	67	53.6	125
Hungary	865	50.7	841	49.3	1706
Ireland	308	54.7	255	45.3	563
Israel	139	58.9	97	41.1	236
Iceland	8	57.1	6	42.9	14
Italy	2028	51.9	1881	48.1	3909
Lithuania	89	47.1	100	52.9	189
Luxembourg	6	60.0	4	40.0	10
The Netherlands	1319	50.2	1306	49.8	2625
Norway	496	56.0	389	44.0	885
Poland	3363	54.8	2774	45.2	6137
Portugal	662	45.1	805	54.9	1467
Russia	5776	50.5	5656	49.5	11 432
Sweden	798	54.7	660	45.3	1458
Slovenia	108	53.5	94	46.5	202
Slovakia	281	52.7	252	47.3	533
Turkey	1285	51.9	1191	48.1	2476
Ukraine	1285	54.1	1089	45.9	2374
Total	44 223	52.8	39 511	47.2	83 734

We analyzed the attitudes to the following three kinds of migrants: (a) immigrants of **S**ame race/**E**thnic group (SE), (b) immigrants of **D**ifferent race/**E**thnic group (DE), and (c) immigrants from **P**oorer **C**ountries outside Europe (PC), via consent to any of the following four questions, encoded as 1 = *allow many to come and live here*, 2 = *allow some to come and live here*, 3 = *allow a few to come and live here*, and *4 = allow none to come and live here*. Additionally, we used the following variables: sex (encoded as 1 = men, 2 = women); age in years from 18 to 60; highest education encoded as 1 = less than lower secondary education, 2 = lower secondary education completed, 3 = upper secondary education completed, 4 = post‐secondary nontertiary education completed, 5 = tertiary education completed; religious intensity measured by the attendance of religious services encoded as 1 = never, 2 = less often, 3 = only on special holydays, 4 = at least once a month, 5 = once a week, 6 = more than once a week, 7 = every day, political orientation measured on a Likert scale from 0 = most left to 10 = most right, and whether or not a child was ever present in the household (0 = never, 1 = ever) as a proxy for childlessness.

We calculated the following general mixed ordinal models using the R library ordinal: regressing (a) SE, (b) DE, and (c) PC on sex, age, education, religious intensity, political orientation, and ever‐presence of a child in the household, with country, survey round, and religious denomination as random factors. We included all one‐way interactions with sex, eliminating interactions by the Akaike information criterion and significance. Additionally, we calculated the odds ratios for each explaining variable, which quantify the effect on the dependent variable (ie, attitude toward migrants) if the explaining variable changes for one unit.

## 3 RESULTS

Albeit countries in the sample differ substantially in their attitude toward migration, overall, the majority of respondents in most of the countries and ESS rounds prefers moderate migration (ie, “allow some to come”) followed by a more restrictive attitude toward migration (ie, “allow a few to come”). However, the proportions differ according to the kind of migrants: a more migration‐friendly attitude toward migration of people from own ethnicity changes to a more restrictive attitude toward migration of people from different ethnicity or from poorer countries (Table 2). Thus, although the preference of a moderate migration holds true for all sorts of migration, the percentage of a more skeptical attitude toward migration increases if migrants have a different ethnic background or come from poorer countries (Table 2). Also on country level, moderate migration (“allow some to come”) is preferred by the majority, followed by restricted migration (“allow a few to come”), even though data for single countries and SSE rounds should be treated with caution, as the number of cases in each group is low (Tables S1‐S3).

**TABLE 2:** Frequencies and percentages of attitude toward migration of same ethnicity, different ethnicity, and from poorer countries

	Same ethnicity		Different ethnicity		Poorer countries	
	N	%	N	%	N	%
Allow many to come and live here	20 101	24.56	12 232	14.94	11 410	13.99
Allow some to come and live here	37 293	45.57	33 849	41.33	31 513	38.65
Allow a few to come and live here	18 592	22.72	25 606	31.27	25 977	31.86
Allow none to come and live here	5852	7.15	10 206	12.46	12 640	15.50
Total	81 838	100.00	81 894	100.00	81 540	100.00

In the multivariate models, we further find that in all three models, being a woman is associated with a friendlier attitude toward migrants than being a man (Tables 3, 4, and 5). The increasingly lower odds ratios indicate that this difference in attitude between men and women increases if migration is from different ethnicity or from poorer countries. In addition, increasing age is associated with increasing skepticism toward migration. Again, odds ratios indicate that this increase is somewhat stronger for migrants from a different ethnic background and from poorer countries (Tables 3, 4, 5). The significant positive interaction between being a women and age further indicates that in women, the critical attitude toward migration increases more steeply with increasing age than in men, which, according to odds ratios, is found irrespective of the type of migration (Tables 3, 4, 5). Higher education is associated with a more migration‐friendly attitude. Odds ratios indicate that this holds particularly true for migrants from a different ethnic background, followed by migrants of the same ethnic background and migrants from poorer countries. Also, being religiously more active is associated with a friendlier attitude toward migration. Here, the slightly decreasing odds ratios indicate a somewhat friendlier attitude toward migration from different ethnicity and poorer countries in religiously more active individuals (Tables 3, 4, 5). Unsurprisingly, having a more “right wing” political orientation is associated with a more restrictive attitude toward migration. According to odds ratios, the restrictive attitude is higher for migration of different ethnic background and migration from poorer countries than migration from same ethnic background. Ever having a child in the household is also associated with a more restrictive attitude but only in the case of migration from a different ethnic background and migration from poorer countries with no substantial difference in odds ratios between these two kinds of migration (Tables 3, 4, 5).

**TABLE 3:** Generalized ordinal mixed model of the attitude toward migrants of the same ethnicity (1 = allow many to come, 4 = allow none to come), regressing on sex, age, highest education, attendance of religious services (1 = never, 7 = every day), political orientation (0 = left, 10 = right) and ever presence of a child in the household (0 = never, 1 = ever), interaction of sex and age, with country, ESS round, and religious denomination as random factors

	Estimate	Standard error	*z* value	*P*	Odds ratio	Confid. 2.5%	Confid. 97.5%
Sex, women (ref. men)	−0.1921	0.0541	−3.5520	.0004	0.8253	0.7423	0.9175
Age at survey	0.0064	0.0011	5.8020	.0000	1.0064	1.0043	1.0086
Highest education	−0.2220	0.0080	−27.8660	.0000	0.8009	0.7885	0.8135
Attendance religious services	−0.0330	0.0073	−4.4930	.0000	0.9676	1.0188	1.0485
Political: Left‐right	0.0574	0.0046	12.3570	.0000	1.0591	1.0495	1.0688
Child in HH (ref. no child)	−0.0164	0.0268	−0.6100	.5417	0.9838	0.9334	1.0368
Sex women (ref. men):Age at survey	0.0043	0.0013	3.3080	.0009	1.0043	1.0017	1.0068
**Number of observations**	**Log likelihood**	**AIC**	**Number of interatios**				
41 790	−48 638.15	97 298.3	1316(7509)				
**Random effects**							
**Groups**	**Name**	**Variance**	**Standard deviation**				
Country:essround:Religious_Denomination	(Intercept)	0.5143	0.7172				
Number of groups	770						
degrees of freedom	82 793						

**TABLE 4:** Generalized ordinal mixed model of the attitude toward migrants of different ethnicity (1 = allow many to come, 4 = allow none to come), regressing on sex, age, highest education, attendance of religious services (1 = never, 7 = every day), political orientation (0 = left, 10 = right), and ever presence of a child in the household (0 = never, 1 = ever), interaction of sex and age, with country, ESS round, and religious denomination as random factors

	Estimate	Standard error	*z* value	*P*	Odds ratio	Confid. 2.5%	Confid. 97.5%
Sex women (ref. men)	−0.2619	0.0540	−4.8510	.0000	0.7696	0.6923	0.8555
Age at survey	0.0090	0.0011	8.0970	.0000	1.0090	1.0068	1.0112
Highest education	−0.2467	0.0080	−30.9100	.0000	0.7814	0.7692	0.7937
Attendance religious services	−0.0411	0.0074	−5.5970	.0000	0.9597	0.9460	0.9736
Political: Left‐right	0.1070	0.0047	22.9540	.0000	1.1129	1.1028	1.1232
Child in HH (ref. no child)	0.0825	0.0269	3.0720	.0021	1.0860	1.0303	1.1447
Sex women (ref. men):Age at survey	0.0046	0.0013	3.5850	.0003	1.0046	1.0021	1.0072
**Number of observations**	**Log likelihood**	**AIC**	**Number of interations**				
41 726	−48 883.51	97 789.03	1444(7230)				
**Random effects**							
**Groups**	**Name**	**Variance**	**Standard deviation**				
Country:essround:Religious_Denomination	(Intercept)	0.6355	0.7972				
Number of groups	769						
degrees of freedom	82 761						

**TABLE 5:** Generalized ordinal mixed model of attitude toward migrants from poorer countries (1 = allow many to come, 4 = allow none to come), regressing on sex, age, highest education, attendance of religious services (1 = never, 7 = every day), political orientation (0 = left, 10 = right), and ever presence of a child in the household (0 = never, 1 = ever), interaction of sex and age, with country, ESS round, and religious denomination as random factors

	Estimate	Standard error	*z* value	*P*	Odds ratio	Confid. 2.5%	Confid. 97.5%
Sex women (ref. men)	−0.3125	0.0538	−5.8040	.0000	0.7316	0.6584	0.8131
Age at survey	0.0100	0.0011	9.0250	.0000	1.0100	1.0078	1.0122
Highest education	−0.1997	0.0079	−25.1860	.0000	0.8189	0.8063	0.8318
Attendance religious services	−0.0556	0.0073	−7.5720	.0000	0.9459	0.9324	0.9596
Political: Left‐right	0.1087	0.0047	23.3540	.0000	1.1148	1.1047	1.1250
Child in HH (ref. no child)	0.0770	0.0268	2.8750	.0040	1.0801	1.0248	1.1383
Sex women (ref. men):Age at survey	0.0051	0.0013	3.9750	.0001	1.0051	1.0026	1.0077
**Number of observations**	**Log likelihood**	**AIC**	**Number of interations**				
41 559	−49 394.93	98 811.85	1429(7044)				
**Random effects**							
**Groups**	**Name**	**Variance**	**Standard deviation**				
Country:essround:Religious_Denomination	(Intercept)	0.7046	0.8394				
Number of groups	771						
degrees of freedom	82 505						

## 4 DISCUSSION

Overall (integrating all countries and rounds of the ESS), we find that the majority of respondents prefer moderate migration (ie, “allow some to come”), particularly if migrants have the same ethnic background. A very open attitude toward migration (“allow many to come”) is only favored by a minority, and this open attitude is declining the more “out‐group” the migrants are (from 24.56% for migrants of the same ethnicity to 14.94% and 13.99% for migrants of different ethnicity and poorer countries, respectively). We further find that men are generally more skeptical toward migration than women. Odds ratios further indicate that this difference between men and women is higher if migrants have different ethnic background or come from poorer countries.

We would argue that these differences in attitudes between men and women may also be rooted in our evolutionary past. The genome—as a chronicler of past sexual encounters—reveals much about past (and particularly prehistoric) human interactions. In Europe, for instance, the main genetic component before the arrival of the Yamnaya consisted of the Anatolian farmers and small populations of ancient hunter gatherers. The arrival of the Yamnaya people in Europe about 5000 years age (Fu et al., 2016; Goldberg et al., 2017; Reich, 2018) dramatically changed the genetic landscape of Europe, particularly the paternal lines of heredity: the proportion of Y‐chromosomes inherited from the residential Anatolian farmers (who entered Europe approximately 8000 years ago) and the remaining ancient hunter gatherer populations (being in Europe since 40.000 years) declined rapidly after the arrival of the male‐biased Yamnaya migration (Goldberg et al., 2017) and even became extinct in some regions (Olalde et al., 2019). Both in Europe and also the Indian subcontinent, the Y‐chromosomal data indicate that the newly arriving migrants mixed with the resident female population, to the massive disadvantage of the local male population, leading to a virtual extinction of the residential male population (described in Reich, 2018). The Yamnaya themselves emerged out of an admixture of two populations some 7000 to 5000 years ago formed through a steady genetic influx from two populations from the south into the Steppe (Ukraine/Russia) (Haak et al., 2015). They grew in size and eventually headed west and also south‐east as far as India. But not only the Yamnaya expansion is an impressive example for a displacement of ancient residential male populations but also the Anglo Saxon invasion in Britain (Weale, Weiss, Jager, Bradman, & Thomas, 2002) and most recently the male dominated colonization of the Americas (Reich et al., 2012).

On the other hand, there are also numerous examples of—presumably—less violent prehistoric migrations, which eventually led to the admixture of different populations in the long run; such as the migration of Anatolian farmers, that led to an almost replacement of the resident hunter‐gatherer population and a very slow admixture among both groups. This rather slow migration of agriculturalist families over a longer period of time from Anatolia to Europe ~8000 years ago (Fu et al., 2016; Goldberg et al., 2017; Nielsen, Akey, Jakobsson, et al., 2017; Skoglund et al., 2012) first resulted in “parallel societies” of resident hunter‐gatherers and agriculturalist but later led to an admixture of both populations. In this case, no evidence of the sex‐specific admixture has been found. However, in both cases, after the Anatolian expansion as well as after the Yamnaya spread, data indicate a resurgence of the genetic representation of the residential inhabitants: during the Middle Neolithic, hunter‐gatherer ancestry rose again after its Early Neolithic decline, and then between the Late Neolithic and the present, when farmer and hunter‐gatherer ancestry rose after its Late Neolithic decline (Haak et al., 2015). Archeological evidence indicates that the rather slow migration wave from Anatolia seems to have been less violent compared to the Yamnaya expansion, but recent data also suggest that violence also has been occurred during the Anatolian expansion (Alt et al., 2020).

From the perspective of evolutionary psychology, however, the examples of violent encounters (Jantzen et al., 2011; Wild et al., 2004) might at least partially explain why men are more skeptical toward migration. In addition, the Y‐chromosomal asymmetric mixing contributes evidence to the long debated question if male aggression and violence would be adaptive or not, in particular, inter‐group aggression and killing (Archer, 2009, Daly, 2015, Macfarlan, Walker, Flinn, & Chagnon, 2014, Glowacki & Wrangham, 2015, reviewed in Wrangham, 2018). Clearly, the invading Yamnaya mended have reproductive and thus evolutionary benefits.

Our data also show that women are not per se more migration friendly compared tome but that in women more than in men, attitude toward migration changes with age. As women grow older, they become increasingly more skeptic of migration, whereas younger women are on average more migration friendly. This “friendliness” in younger women might be interpreted in the light of female dispersal (Huber, Zahourek, & Fieder, 2017). In the Paleolithic, hunter‐gatherer mating networks (Sikora et al., 2017) may have existed, which may have helped to avoid inbreeding by “marriage migration” between groups. Particularly, young women may have left their natal group to live with their husband's family (Seielstad, Minch, & Cavalli‐Sforza, 1998; Sterck, 1998; Towner, 2002), a scenario, where friendliness toward strangers would probably be advantageous. In addition, in the case of hostile group encounters, women have faced a substantially lower danger of being injured or killed (Wrangham, 2018) which was particularly true if they had a more out‐group friendly attitude. Furthermore, openness toward strangers may have fostered cultural exchange between groups that in terms of acquired and transmitted practices and techniques may have been advantageous for survival in hostile environments (Henrich, 2015). The finding that women may become more skeptical toward migration with increasing age, may also be interpreted on the basis of theory on general intelligence with a ability to learn new information and seek novel experiences at younger ages and a later life combination of these information and experiences to a “crystallized knowledge” (Geary, 2004). These findings indicate that selection may be acting at least to some extent antagonistically in men and in women (Connallon, Cox, & Calsbeek, 2010).

Overall, increasing age is associated with a more skeptical attitude toward migrants in both men and women. Although odds ratios indicate that the increase per year of life is rather small, over the whole life span, this effect accumulates to result in a substantial shift from a more positive to a more skeptic attitude. This finding is in line with the literature on changes of attitudes during the course of life (Visser & Krosnick, 1998).

The finding that having at least one child in the household (a rough proxy of ever reproducing) is associated with a more skeptical attitude toward migration of people of different ethnicity and from poorer countries indicates that individuals who have reproduced have a more critical attitude toward some kinds of migration compared to never reproducing individuals. We can only speculate why this is the case. Maybe parents are worrying for the future of their progeny, if the social tensions that are at times associated with migration are taken into account.

Our data further show that two parameters may have the potential to lower skepticisms toward migration: education and religiousness. Higher education is not only associated with a positive attitude toward migration, in particular toward migration of different ethnic background. Indicated by the odds ratios, the size of the effect of education, increasing from the lowest to the highest education level and leading to a higher acceptance of migrants particularly from a different culture, is comparable to that of sex. This migration‐friendly attitude may be interpreted as an effect of education per se, as education particularly in western societies is usually strong emphasizing mutual understanding and tolerance (Craft, 2017). However, individuals with higher education are usually better off in terms of resources compared to less educated people. As a result, higher educated individuals are usually less directly affected by migration, for instance, in terms of competition on the job or housing market (Collier, 2013).

Also religiousness is associated with a more positive attitude toward migration. Odds ratios indicate that over the full scale, religiousness has a comparable effect size as sex and education. This more positive attitude may be interpreted by the charitable characteristics of religion and the highly inclusive potential of some religions (Huber & Fieder, 2018; Norenzayan, 2013). This view is supported by the finding that the positive attitude is even higher if migrants have different ethnic background or come from poorer countries. Hence, this finding could be an indication for the integrative power of some religions even though religions also have a clearly excluding character, leading to intergroup conflicts (Seul, 1999). Nonetheless, the tendency to integrate individuals from different ethnic background maybe interpreted by the integrative power of religions during the agricultural revolution—when people with a different ethnic and cultural background settled into larger agglomerations (Norenzayan et al., 2016)—as well as inherent moral dogmas. However, our sample is by far mostly from predominantly “Christian countries.” In the models we calculated separately for the only predominantly Muslim country in our sample “Turkey” (only Muslims, parents born in Turkey and no experience of discrimination reported), we found no significant association between religious intensity and attitude to migrants whatsoever (data not shown).

As expected, a more right wing political attitude is associated with a more restrictive attitude toward migrants, particularly if migrants have a different ethnicity or are from poorer countries. As political attitude is encoded on an 11‐item scale, over the total range of the scale, despite rather small odds ratios, the association of the political attitude with the attitude toward migration is substantial. An association between right wing orientation and skepticism toward migration has also been demonstrated by Hatemi and McDermott (2012). Also, twin studies have shown that political attitude has a genetic basis (Hatemi & McDermott 2012) and in‐group favoritism has in part a genetic basis that may contribute to this trait. The variance in “in‐group favoritism” explained by inheritance in twin studies is varying greatly from 18% to 79% depending on the actual trait surveyed and how the in‐group has been defined (Kandler, Lewis, Feldhaus, & Riemann, 2015; Lewis, Kandler, & Riemann, 2014; Loehlin, 1993). Constructing a sum indicator of all three questions on the attitudes toward migrants and regressing political attitude on this indicator, attitude toward migrants explains about 16% of the variance in political attitude of the survey participants. Hence, as expected, the attitude toward strangers and political orientation is associated and explains a certain proportion of variance. Overall, we assume that political orientation is a trait that evolved in an interplay between genes and the environment (Alford et al., 2011; Hatemi & McDermott, 2012), that is, as a process of a cultural genetic coevolution (Richerson, Boyd, & Henrich, 2010). As political orientation has a rather strong genetic basis, both left wing and right wing political orientation should have brought benefits to the bearer from an evolutionary point of view. Accordingly, political orientation (both left and right) is related to the number of offspring, indicating reproductive advantages for the political extreme (Fieder & Huber, 2018). However, this pattern shifted in Western industrial countries to only a reproductive advantage for the political right (Fieder & Huber, 2018). In line with Stearns, Byars, Govindaraju, and Ewbank (2010), it can be assumed that this reproductive differential maybe suitable to indicate selective forces for political orientation.

The overall more reluctant attitude to migrants of a different ethnicity compared with the attitude toward migrants of the same ethnicity may indicate some sort of ethnic nepotism toward more similar individuals (Rushton, 1989, Salter & Harpeding, 2013), thus humans may have preferred individuals where they detected genetic similarity and that such a behavior may have overall enhanced fitness (Rushton et al., 1985, Rushton, 1989, Salter & Harpeding, 2013). On basis of data from 183 countries worldwide, Vanhanen found evidence that ethnic division tends to lead to conflicts of interest between ethnic groups and that the more a society is ethnically divided, the more political and other conflicts arise along ethnic line (Vanhanen, 1999), as well as intensities along religious borders (Salter, 2018). Also, ethnic nepotism maybe adaptive if it helps to secure resources and territory (Salter, 2002). Furthermore, religious and ethnic homogamy increases the average number of children and decreases childlessness (Fieder & Huber, 2016b; Huber & Fieder, 2018). It has been demonstrated on the basis of data from Iceland, that average offspring number decreases with genetic relatedness from second‐order cousins on (Helgason, Pálsson, Guðbjartsson, & Stefánsson, 2008) and thus during our evolutionary past moderate inbreeding may have led to reproductive benefits (Fox, 2015).

Ethnic nepotism may have some serious drawbacks, as individuals may become overall—or for certain traits—more homozygote with all the known consequences (Clark et al., 2019, Fieder, Huber, & Martin, n.d., submitted). Particularly in small‐scale societies of mostly not more than 150 individuals (Dunbar, 1993), the burden of inbreeding may have been large in the case of ethnic nepotism. Ethnic nepotism and the rejection of strangers, as well as a more open attitude toward strangers, always depend on the actions of others. Therefore, such an adopted strategy may lead to an evolutionary stable strategy under certain conditions (Smith, 1976) with different characteristics within a group. Ethnic nepotism has no concept of “ethnic kinship” (such as kinship between parents and children) that would make this more distant “altruism” reasonable. A way out of this dilemma has been proposed by Jones (2018), indicating that ethnic is nepotism expressed by a group toward their own kin and thus individuals of similar phenotypes. In‐group marriages in small groups fostered genetic relatedness also among individuals that are more distantly related than close kin and thus in‐group cooperation among individuals that are genetically more related as indicated by simple kinship. An argument quite closely related to Hamilton (Hamilton, 1964, 1971) and West et al. (2011). Accordingly, William D. Hamilton also argued that successfully expanding groups are expected to evolve to some extent a degree of xenophobia (Hamilton, 1996). But ethnic nepotism may have resulted in high levels of inbreeding with all the related consequences and thus a process of balancing selection may have been beneficial: some individuals with a propensity for an increased out‐group cooperation.

Thus we assume that balancing selection may help to explain the attitudes toward strangers: both a more open attitude as well as a more guarded attitude may have led to benefits for the bearer, and so both attitudes have been kept in the population (Charlesworth, 2006; Fieder & Huber, 2018)—presumably at different frequencies, depending on the environment and circumstances. Evidence for a balancing selection on the “attitudes to strangers” maybe found in the polymorphisms of alleles that are associated with this attitude. If balancing selection would have been acting on alleles associated with attitudes toward strangers, balancing selection may have increased the polymorphism of loci that are associated with the trait “attitude toward strangers”. We are not aware of any GWA study that already identified loci associated with the attitude toward in‐group vs out‐group members; hence, this argument remains speculative at the moment. One hint for balancing selection is evidence from the World Value Survey: as in the case of political attitude, individuals with a more critical attitude toward strangers have on average more children (data not published). Balancing selection may explain different attitudes toward strangers, while differences between men and women may be additionally explained by a “sex‐specific selection”. The same trait may lead to different reproductive outcomes for men and women and thus for different selection pressures on the sexes (Connallon et al., 2010).

Our data set provides no information on the attitude toward strangers and pathogen prevalence, but pathogens may have also been important in shaping our attitude toward strangers. Infectious diseases clearly increased morbidity and mortality throughout human history; this may particularly hold true since the rise of agricultural societies and thus more dense human populations (Wolfe, Dunavan, & Diamond, 2007). Previous research demonstrated worldwide variability in pathogen prevalence, predicting some cultural differences ranging from food habits (Sherman & Billing, 1999), marriage structures (Low, 1990), parenting practices (Quinlan, 2007), and mate choice (Gangestad & Buss, 1993). Interestingly, it seems that attitudes of collectivism (eg, ethnocentrism, conformity) can inhibit the transmission of pathogens. Fincher, Thornhill, Murray, and Schaller (2008) demonstrated on the basis of worldwide data that the regional prevalence of pathogens correlates strongly positive with cultural indicators of collectivism but strongly negative with individualism. However, admixture and thus heterozygosity may help to cope better with diseases and infections, as heterozygosity seems to improve also the health condition of individuals and thus may increase protection against pathogens (Clark et al., 2019). This may especially hold true for major histocompatibility complex heterozygosity (Penn, Damjanovich, & Potts, 2002; Xu et al., 2019).

From an evolutionary point of view, a more negative attitude toward migrants and a positive attitude toward migrants make sense: In some cases, migration may include violence with detrimental outcomes for certain groups (particularly men). On the other hand, isolation may lead to lack of cultural exchange and to an increase in inbreeding.^1^Considering the size of populations in contemporary Europe, inbreeding avoidance has to be considered an evolutionary inherited trait as it has limited relevance today. Also, the necessity of cultural exchange to thrive (Henrich, 2015) has been altered by contemporary technology allowing global communication and information exchange.

Our findings are also in line with Eibl‐Eibesfeldt, who suggested that with regard to strangers, humans are “an ambivalent species,” displaying both timidity and interest—particularly in the case of recurring interactions (Eibl‐Eibesfeldt, 1986). Thus, in our evolutionary history, both traits may have been crucial for survival and we assume that both—evolutionary acquired—predispositions are still present in contemporary populations and influence the perception of migration flows. Migration‐friendly vs migration‐skeptical attitude may serve as an example of a genetic cultural coevolution (Henrich, 2015; Richerson et al., 2010). As our data are based on European populations, our conclusions are, of course, limited to this group.

To conclude, we would like to stress that it is of utmost importance to note that evolutionary acquired mind‐sets are not a predicament. Human behavior has in many ways improved during the history of our species, taming detrimental instincts and making us “better angels or our nature” as Steven Pinker put it (Pinker, 2011).

## CONFLICT OF INTEREST

The authors declare no potential conflict of interest.

## AUTHOR CONTRIBUTIONS

A.S. interpreted the data and wrote the manuscript. S.H. interpreted the data and wrote the manuscript. M. F. conducted the analyses, interpreted the data, and wrote the manuscript. All authors read and approved the final manuscript.

## Supporting information


**Table S1** Same ethnicity
**Table S2** Different ethnicity
**Table S3** Poorer countries

## Data Availability

All raw data used in the manuscript can be downloaded after registration from the “European Social Survey” at: https://www.europeansocialsurvey.org/data/conditions_of_use.html.

## Endnote

1 It has to be noted that albeit inbreeding may have detrimental health effects, an increased number of offspring for couples related at the level of third‐ and fourth‐order cousins indicate that moderate inbreeding may in fact have fitness benefits (Helgason et al., 2008). Also, as outbreeding may avoid the detrimental genetic effects of inbreeding and consanguinity, it may induce costs: outbreeding with genetically very distant individuals may, for instance, lead to breaking up of advantageous allelic combinations (adaptive alleles—Jobling et al., 2013) and thus to reduced fitness under certain environmental conditions. Hence, an “optimal outbreeding” may exist when individuals choose mates minimizing the inclusive fitness costs of both inbreeding and outbreeding (Durham, 1991, pp 331‐339).
