# Supplementary material for: Factors affecting attitudes toward migrants—An evolutionary approach
Source: Am J Hum Biol. 2020 May 26;33(1):e23435. doi: 10.1002/ajhb.23435 (PMC7900986; doi:10.1002/ajhb.23435)
Supplement: Supplementary file 1 — Table S1 Same ethnicity Table S2 Different ethnicity Table S3 Poorer countries [file AJHB-33-e23435-s001.docx]

Table S1 Same Ethnicity

| **Allow Migrants Same Ethinicity** |  |  |  |  |  |  |  |  |  |  |  |
| --- | --- | --- | --- | --- | --- | --- | --- | --- | --- | --- | --- |
| Country |  |  | ESS round |  |  |  |  |  |  |  | Total |
|  |  |  | 1 | 2 | 3 | 4 | 5 | 6 | 7 | 8 |  |
| Austria | Allow many to come and live here | N | 16 | 32 | 36 |  |  |  | 40 | 31 | 155 |
|  |  | % in ESS round | 12.1% | 20.4% | 20.3% |  |  |  | 19.6% | 16.5% | 18.1% |
|  | Allow some | N | 55 | 80 | 81 |  |  |  | 90 | 84 | 390 |
|  |  | % in ESS round | 41.7% | 51.0% | 45.8% |  |  |  | 44.1% | 44.7% | 45.5% |
|  | Allow a few | N | 55 | 37 | 51 |  |  |  | 58 | 53 | 254 |
|  |  | % in ESS round | 41.7% | 23.6% | 28.8% |  |  |  | 28.4% | 28.2% | 29.6% |
|  | Allow none | N | 6 | 8 | 9 |  |  |  | 16 | 20 | 59 |
|  |  | % in ESS round | 4.5% | 5.1% | 5.1% |  |  |  | 7.8% | 10.6% | 6.9% |
|  |  | N | 132 | 157 | 177 |  |  |  | 204 | 188 | 858 |
|  |  | % in ESS round | 100.0% | 100.0% | 100.0% |  |  |  | 100.0% | 100.0% | 100.0% |
| Belgium | Allow many to come and live here | N | 24 | 47 | 37 | 46 | 35 | 33 | 35 | 62 | 319 |
|  |  | % in ESS round | 12.8% | 20.2% | 17.9% | 21.0% | 16.5% | 15.0% | 16.6% | 30.0% | 18.8% |
|  | Allow some | N | 107 | 116 | 119 | 119 | 113 | 134 | 118 | 107 | 933 |
|  |  | % in ESS round | 57.2% | 49.8% | 57.5% | 54.3% | 53.3% | 60.9% | 55.9% | 51.7% | 55.0% |
|  | Allow a few | N | 43 | 51 | 37 | 41 | 45 | 41 | 42 | 31 | 331 |
|  |  | % in ESS round | 23.0% | 21.9% | 17.9% | 18.7% | 21.2% | 18.6% | 19.9% | 15.0% | 19.5% |
|  | Allow none | N | 13 | 19 | 14 | 13 | 19 | 12 | 16 | 7 | 113 |
|  |  | % in ESS round | 7.0% | 8.2% | 6.8% | 5.9% | 9.0% | 5.5% | 7.6% | 3.4% | 6.7% |
|  |  | N | 187 | 233 | 207 | 219 | 212 | 220 | 211 | 207 | 1696 |
|  |  | % in ESS round | 100.0% | 100.0% | 100.0% | 100.0% | 100.0% | 100.0% | 100.0% | 100.0% | 100.0% |
| Bulgaria | Allow many to come and live here | N |  |  | 45 | 48 | 55 | 44 |  |  | 192 |
|  |  | % in ESS round |  |  | 51.1% | 48.0% | 48.2% | 38.9% |  |  | 46.3% |
|  | Allow some | N |  |  | 26 | 34 | 37 | 42 |  |  | 139 |
|  |  | % in ESS round |  |  | 29.5% | 34.0% | 32.5% | 37.2% |  |  | 33.5% |
|  | Allow a few | N |  |  | 12 | 13 | 13 | 17 |  |  | 55 |
|  |  | % in ESS round |  |  | 13.6% | 13.0% | 11.4% | 15.0% |  |  | 13.3% |
|  | Allow none | N |  |  | 5 | 5 | 9 | 10 |  |  | 29 |
|  |  | % in ESS round |  |  | 5.7% | 5.0% | 7.9% | 8.8% |  |  | 7.0% |
|  |  | N |  |  | 88 | 100 | 114 | 113 |  |  | 415 |
|  |  | % in ESS round |  |  | 100.0% | 100.0% | 100.0% | 100.0% |  |  | 100.0% |
| Switzerland | Allow many to come and live here | N | 26 | 31 | 37 | 33 | 33 | 28 | 28 | 31 | 247 |
|  |  | % in ESS round | 20.3% | 21.1% | 27.8% | 23.7% | 25.0% | 19.3% | 21.5% | 24.6% | 22.9% |
|  | Allow some | N | 77 | 92 | 74 | 83 | 79 | 94 | 84 | 72 | 655 |
|  |  | % in ESS round | 60.2% | 62.6% | 55.6% | 59.7% | 59.8% | 64.8% | 64.6% | 57.1% | 60.6% |
|  | Allow a few | N | 24 | 20 | 20 | 19 | 18 | 21 | 16 | 20 | 158 |
|  |  | % in ESS round | 18.8% | 13.6% | 15.0% | 13.7% | 13.6% | 14.5% | 12.3% | 15.9% | 14.6% |
|  | Allow none | N | 1 | 4 | 2 | 4 | 2 | 2 | 2 | 3 | 20 |
|  |  | % in ESS round | .8% | 2.7% | 1.5% | 2.9% | 1.5% | 1.4% | 1.5% | 2.4% | 1.9% |
|  |  | N | 128 | 147 | 133 | 139 | 132 | 145 | 130 | 126 | 1080 |
|  |  | % in ESS round | 100.0% | 100.0% | 100.0% | 100.0% | 100.0% | 100.0% | 100.0% | 100.0% | 100.0% |
| Cyprus | Allow many to come and live here | N |  |  | 2 | 7 | 4 | 2 |  |  | 15 |
|  |  | % in ESS round |  |  | 12.5% | 36.8% | 28.6% | 14.3% |  |  | 23.8% |
|  | Allow some | N |  |  | 6 | 8 | 3 | 3 |  |  | 20 |
|  |  | % in ESS round |  |  | 37.5% | 42.1% | 21.4% | 21.4% |  |  | 31.7% |
|  | Allow a few | N |  |  | 7 | 3 | 5 | 6 |  |  | 21 |
|  |  | % in ESS round |  |  | 43.8% | 15.8% | 35.7% | 42.9% |  |  | 33.3% |
|  | Allow none | N |  |  | 1 | 1 | 2 | 3 |  |  | 7 |
|  |  | % in ESS round |  |  | 6.3% | 5.3% | 14.3% | 21.4% |  |  | 11.1% |
|  |  | N |  |  | 16 | 19 | 14 | 14 |  |  | 63 |
|  |  | % in ESS round |  |  | 100.0% | 100.0% | 100.0% | 100.0% |  |  | 100.0% |
| Czechia | Allow many to come and live here | N | 17 | 19 |  | 18 | 34 | 16 | 18 | 19 | 141 |
|  |  | % in ESS round | 10.3% | 11.9% |  | 7.7% | 13.4% | 8.7% | 7.5% | 5.8% | 9.0% |
|  | Allow some | N | 84 | 68 |  | 99 | 93 | 65 | 97 | 111 | 617 |
|  |  | % in ESS round | 50.9% | 42.8% |  | 42.1% | 36.8% | 35.5% | 40.4% | 33.7% | 39.5% |
|  | Allow a few | N | 57 | 56 |  | 96 | 95 | 73 | 84 | 141 | 602 |
|  |  | % in ESS round | 34.5% | 35.2% |  | 40.9% | 37.5% | 39.9% | 35.0% | 42.9% | 38.5% |
|  | Allow none | N | 7 | 16 |  | 22 | 31 | 29 | 41 | 58 | 204 |
|  |  | % in ESS round | 4.2% | 10.1% |  | 9.4% | 12.3% | 15.8% | 17.1% | 17.6% | 13.0% |
|  |  | N | 165 | 159 |  | 235 | 253 | 183 | 240 | 329 | 1564 |
|  |  | % in ESS round | 100.0% | 100.0% |  | 100.0% | 100.0% | 100.0% | 100.0% | 100.0% | 100.0% |
| Germany | Allow many to come and live here | N | 411 | 434 | 407 | 698 | 534 | 697 | 797 | 780 | 4758 |
|  |  | % in ESS round | 21.3% | 23.3% | 21.5% | 36.0% | 29.0% | 38.9% | 43.6% | 41.6% | 31.8% |
|  | Allow some | N | 1015 | 927 | 911 | 930 | 971 | 899 | 877 | 920 | 7450 |
|  |  | % in ESS round | 52.7% | 49.8% | 48.2% | 47.9% | 52.8% | 50.1% | 48.0% | 49.0% | 49.8% |
|  | Allow a few | N | 445 | 387 | 464 | 258 | 283 | 176 | 126 | 170 | 2309 |
|  |  | % in ESS round | 23.1% | 20.8% | 24.6% | 13.3% | 15.4% | 9.8% | 6.9% | 9.1% | 15.4% |
|  | Allow none | N | 55 | 113 | 108 | 55 | 51 | 21 | 28 | 7 | 438 |
|  |  | % in ESS round | 2.9% | 6.1% | 5.7% | 2.8% | 2.8% | 1.2% | 1.5% | .4% | 2.9% |
|  |  | N | 1926 | 1861 | 1890 | 1941 | 1839 | 1793 | 1828 | 1877 | 14955 |
|  |  | % in ESS round | 100.0% | 100.0% | 100.0% | 100.0% | 100.0% | 100.0% | 100.0% | 100.0% | 100.0% |
| Denmark | Allow many to come and live here | N | 30 | 39 | 35 | 41 | 33 | 31 | 33 |  | 242 |
|  |  | % in ESS round | 21.9% | 28.5% | 29.2% | 33.3% | 28.0% | 24.0% | 25.6% |  | 27.1% |
|  | Allow some | N | 76 | 71 | 69 | 65 | 67 | 75 | 71 |  | 494 |
|  |  | % in ESS round | 55.5% | 51.8% | 57.5% | 52.8% | 56.8% | 58.1% | 55.0% |  | 55.3% |
|  | Allow a few | N | 30 | 26 | 13 | 16 | 16 | 21 | 23 |  | 145 |
|  |  | % in ESS round | 21.9% | 19.0% | 10.8% | 13.0% | 13.6% | 16.3% | 17.8% |  | 16.2% |
|  | Allow none | N | 1 | 1 | 3 | 1 | 2 | 2 | 2 |  | 12 |
|  |  | % in ESS round | .7% | .7% | 2.5% | .8% | 1.7% | 1.6% | 1.6% |  | 1.3% |
|  |  | N | 137 | 137 | 120 | 123 | 118 | 129 | 129 |  | 893 |
|  |  | % in ESS round | 100.0% | 100.0% | 100.0% | 100.0% | 100.0% | 100.0% | 100.0% |  | 100.0% |
| Estonia | Allow many to come and live here | N |  | 4 | 3 | 4 | 6 | 6 | 4 | 5 | 32 |
|  |  | % in ESS round |  | 23.5% | 20.0% | 21.1% | 30.0% | 30.0% | 26.7% | 22.7% | 25.0% |
|  | Allow some | N |  | 7 | 7 | 9 | 9 | 9 | 9 | 11 | 61 |
|  |  | % in ESS round |  | 41.2% | 46.7% | 47.4% | 45.0% | 45.0% | 60.0% | 50.0% | 47.7% |
|  | Allow a few | N |  | 5 | 4 | 5 | 4 | 4 | 2 | 5 | 29 |
|  |  | % in ESS round |  | 29.4% | 26.7% | 26.3% | 20.0% | 20.0% | 13.3% | 22.7% | 22.7% |
|  | Allow none | N |  | 1 | 1 | 1 | 1 | 1 | 0 | 1 | 6 |
|  |  | % in ESS round |  | 5.9% | 6.7% | 5.3% | 5.0% | 5.0% | 0.0% | 4.5% | 4.7% |
|  |  | N |  | 17 | 15 | 19 | 20 | 20 | 15 | 22 | 128 |
|  |  | % in ESS round |  | 100.0% | 100.0% | 100.0% | 100.0% | 100.0% | 100.0% | 100.0% | 100.0% |
| Spain | Allow many to come and live here | N | 168 | 197 | 168 | 111 | 222 | 283 | 285 | 322 | 1756 |
|  |  | % in ESS round | 21.9% | 21.3% | 17.3% | 12.4% | 20.2% | 29.4% | 30.8% | 38.5% | 23.8% |
|  | Allow some | N | 287 | 365 | 395 | 294 | 447 | 361 | 367 | 292 | 2808 |
|  |  | % in ESS round | 37.4% | 39.4% | 40.7% | 32.9% | 40.7% | 37.5% | 39.6% | 34.9% | 38.0% |
|  | Allow a few | N | 283 | 312 | 307 | 377 | 324 | 255 | 213 | 191 | 2262 |
|  |  | % in ESS round | 36.8% | 33.7% | 31.6% | 42.2% | 29.5% | 26.5% | 23.0% | 22.8% | 30.6% |
|  | Allow none | N | 30 | 52 | 101 | 112 | 106 | 64 | 61 | 32 | 558 |
|  |  | % in ESS round | 3.9% | 5.6% | 10.4% | 12.5% | 9.6% | 6.6% | 6.6% | 3.8% | 7.6% |
|  |  | N | 768 | 926 | 971 | 894 | 1099 | 963 | 926 | 837 | 7384 |
|  |  | % in ESS round | 100.0% | 100.0% | 100.0% | 100.0% | 100.0% | 100.0% | 100.0% | 100.0% | 100.0% |
| Finland | Allow many to come and live here | N | 20 | 21 | 20 | 27 | 16 | 24 | 25 | 31 | 184 |
|  |  | % in ESS round | 13.7% | 15.2% | 13.1% | 18.8% | 12.0% | 17.9% | 20.3% | 23.0% | 16.6% |
|  | Allow some | N | 69 | 65 | 78 | 72 | 67 | 70 | 65 | 71 | 557 |
|  |  | % in ESS round | 47.3% | 47.1% | 51.0% | 50.0% | 50.4% | 52.2% | 52.8% | 52.6% | 50.4% |
|  | Allow a few | N | 53 | 49 | 54 | 43 | 47 | 39 | 32 | 31 | 348 |
|  |  | % in ESS round | 36.3% | 35.5% | 35.3% | 29.9% | 35.3% | 29.1% | 26.0% | 23.0% | 31.5% |
|  | Allow none | N | 4 | 3 | 1 | 2 | 3 | 1 | 1 | 2 | 17 |
|  |  | % in ESS round | 2.7% | 2.2% | .7% | 1.4% | 2.3% | .7% | .8% | 1.5% | 1.5% |
|  |  | N | 146 | 138 | 153 | 144 | 133 | 134 | 123 | 135 | 1106 |
|  |  | % in ESS round | 100.0% | 100.0% | 100.0% | 100.0% | 100.0% | 100.0% | 100.0% | 100.0% | 100.0% |
| France | Allow many to come and live here | N | 135 | 155 | 150 | 128 | 145 | 183 | 147 | 249 | 1292 |
|  |  | % in ESS round | 13.6% | 15.2% | 14.2% | 11.9% | 13.6% | 18.9% | 19.9% | 25.1% | 16.3% |
|  | Allow some | N | 544 | 570 | 567 | 631 | 596 | 550 | 400 | 536 | 4394 |
|  |  | % in ESS round | 54.7% | 55.7% | 53.6% | 58.7% | 56.1% | 56.8% | 54.1% | 53.9% | 55.5% |
|  | Allow a few | N | 252 | 240 | 285 | 263 | 249 | 213 | 164 | 144 | 1810 |
|  |  | % in ESS round | 25.3% | 23.5% | 27.0% | 24.5% | 23.4% | 22.0% | 22.2% | 14.5% | 22.9% |
|  | Allow none | N | 64 | 58 | 55 | 53 | 73 | 22 | 28 | 65 | 418 |
|  |  | % in ESS round | 6.4% | 5.7% | 5.2% | 4.9% | 6.9% | 2.3% | 3.8% | 6.5% | 5.3% |
|  |  | N | 995 | 1023 | 1057 | 1075 | 1063 | 968 | 739 | 994 | 7914 |
|  |  | % in ESS round | 100.0% | 100.0% | 100.0% | 100.0% | 100.0% | 100.0% | 100.0% | 100.0% | 100.0% |
| United Kingdom | Allow many to come and live here | N | 149 | 108 | 100 | 121 | 116 | 102 | 138 | 202 | 1036 |
|  |  | % in ESS round | 13.2% | 11.8% | 10.4% | 11.0% | 12.2% | 11.5% | 15.6% | 20.6% | 13.3% |
|  | Allow some | N | 606 | 480 | 535 | 613 | 462 | 496 | 461 | 536 | 4189 |
|  |  | % in ESS round | 53.7% | 52.5% | 55.6% | 55.9% | 48.7% | 55.9% | 52.3% | 54.6% | 53.7% |
|  | Allow a few | N | 297 | 269 | 272 | 292 | 265 | 238 | 200 | 202 | 2035 |
|  |  | % in ESS round | 26.3% | 29.4% | 28.3% | 26.6% | 28.0% | 26.8% | 22.7% | 20.6% | 26.1% |
|  | Allow none | N | 77 | 58 | 55 | 70 | 105 | 52 | 83 | 41 | 541 |
|  |  | % in ESS round | 6.8% | 6.3% | 5.7% | 6.4% | 11.1% | 5.9% | 9.4% | 4.2% | 6.9% |
|  |  | N | 1129 | 915 | 962 | 1096 | 948 | 888 | 882 | 981 | 7801 |
|  |  | % in ESS round | 100.0% | 100.0% | 100.0% | 100.0% | 100.0% | 100.0% | 100.0% | 100.0% | 100.0% |
| Greece | Allow many to come and live here | N | 14 | 29 |  | 37 | 48 |  |  |  | 128 |
|  |  | % in ESS round | 9.5% | 15.6% |  | 17.3% | 26.2% |  |  |  | 17.5% |
|  | Allow some | N | 31 | 39 |  | 50 | 42 |  |  |  | 162 |
|  |  | % in ESS round | 21.1% | 21.0% |  | 23.4% | 23.0% |  |  |  | 22.2% |
|  | Allow a few | N | 85 | 94 |  | 99 | 58 |  |  |  | 336 |
|  |  | % in ESS round | 57.8% | 50.5% |  | 46.3% | 31.7% |  |  |  | 46.0% |
|  | Allow none | N | 17 | 24 |  | 28 | 35 |  |  |  | 104 |
|  |  | % in ESS round | 11.6% | 12.9% |  | 13.1% | 19.1% |  |  |  | 14.2% |
|  |  | N | 147 | 186 |  | 214 | 183 |  |  |  | 730 |
|  |  | % in ESS round | 100.0% | 100.0% |  | 100.0% | 100.0% |  |  |  | 100.0% |
| Croatia | Allow many to come and live here | N |  |  |  | 18 | 18 |  |  |  | 36 |
|  |  | % in ESS round |  |  |  | 29.5% | 30.5% |  |  |  | 30.0% |
|  | Allow some | N |  |  |  | 26 | 20 |  |  |  | 46 |
|  |  | % in ESS round |  |  |  | 42.6% | 33.9% |  |  |  | 38.3% |
|  | Allow a few | N |  |  |  | 14 | 16 |  |  |  | 30 |
|  |  | % in ESS round |  |  |  | 23.0% | 27.1% |  |  |  | 25.0% |
|  | Allow none | N |  |  |  | 3 | 5 |  |  |  | 8 |
|  |  | % in ESS round |  |  |  | 4.9% | 8.5% |  |  |  | 6.7% |
|  |  | N |  |  |  | 61 | 59 |  |  |  | 120 |
|  |  | % in ESS round |  |  |  | 100.0% | 100.0% |  |  |  | 100.0% |
| Hungary | Allow many to come and live here | N | 36 | 47 | 37 | 61 | 81 | 38 | 44 | 37 | 381 |
|  |  | % in ESS round | 23.4% | 23.7% | 21.0% | 29.5% | 34.6% | 17.5% | 18.6% | 16.5% | 23.1% |
|  | Allow some | N | 51 | 63 | 59 | 78 | 78 | 68 | 79 | 64 | 540 |
|  |  | % in ESS round | 33.1% | 31.8% | 33.5% | 37.7% | 33.3% | 31.3% | 33.3% | 28.6% | 32.8% |
|  | Allow a few | N | 57 | 63 | 56 | 52 | 56 | 85 | 80 | 47 | 496 |
|  |  | % in ESS round | 37.0% | 31.8% | 31.8% | 25.1% | 23.9% | 39.2% | 33.8% | 21.0% | 30.1% |
|  | Allow none | N | 10 | 25 | 24 | 16 | 19 | 26 | 34 | 76 | 230 |
|  |  | % in ESS round | 6.5% | 12.6% | 13.6% | 7.7% | 8.1% | 12.0% | 14.3% | 33.9% | 14.0% |
|  |  | N | 154 | 198 | 176 | 207 | 234 | 217 | 237 | 224 | 1647 |
|  |  | % in ESS round | 100.0% | 100.0% | 100.0% | 100.0% | 100.0% | 100.0% | 100.0% | 100.0% | 100.0% |
| Ireland | Allow many to come and live here | N | 16 | 23 | 13 | 13 | 11 | 16 | 11 | 13 | 116 |
|  |  | % in ESS round | 25.8% | 33.3% | 22.8% | 18.3% | 13.6% | 20.0% | 16.7% | 19.1% | 20.9% |
|  | Allow some | N | 35 | 31 | 34 | 36 | 38 | 38 | 27 | 30 | 269 |
|  |  | % in ESS round | 56.5% | 44.9% | 59.6% | 50.7% | 46.9% | 47.5% | 40.9% | 44.1% | 48.6% |
|  | Allow a few | N | 10 | 12 | 8 | 15 | 21 | 19 | 23 | 19 | 127 |
|  |  | % in ESS round | 16.1% | 17.4% | 14.0% | 21.1% | 25.9% | 23.8% | 34.8% | 27.9% | 22.9% |
|  | Allow none | N | 1 | 3 | 2 | 7 | 11 | 7 | 5 | 6 | 42 |
|  |  | % in ESS round | 1.6% | 4.3% | 3.5% | 9.9% | 13.6% | 8.8% | 7.6% | 8.8% | 7.6% |
|  |  | N | 62 | 69 | 57 | 71 | 81 | 80 | 66 | 68 | 554 |
|  |  | % in ESS round | 100.0% | 100.0% | 100.0% | 100.0% | 100.0% | 100.0% | 100.0% | 100.0% | 100.0% |
| Israel | Allow many to come and live here | N | 8 |  |  | 10 | 16 | 19 | 28 | 25 | 106 |
|  |  | % in ESS round | 50.0% |  |  | 38.5% | 45.7% | 38.0% | 50.9% | 56.8% | 46.9% |
|  | Allow some | N | 3 |  |  | 6 | 9 | 9 | 13 | 11 | 51 |
|  |  | % in ESS round | 18.8% |  |  | 23.1% | 25.7% | 18.0% | 23.6% | 25.0% | 22.6% |
|  | Allow a few | N | 3 |  |  | 7 | 5 | 7 | 8 | 5 | 35 |
|  |  | % in ESS round | 18.8% |  |  | 26.9% | 14.3% | 14.0% | 14.5% | 11.4% | 15.5% |
|  | Allow none | N | 2 |  |  | 3 | 5 | 15 | 6 | 3 | 34 |
|  |  | % in ESS round | 12.5% |  |  | 11.5% | 14.3% | 30.0% | 10.9% | 6.8% | 15.0% |
|  |  | N | 16 |  |  | 26 | 35 | 50 | 55 | 44 | 226 |
|  |  | % in ESS round | 100.0% |  |  | 100.0% | 100.0% | 100.0% | 100.0% | 100.0% | 100.0% |
| Iceland | Allow many to come and live here | N |  | 2 |  |  |  | 3 |  | 3 | 8 |
|  |  | % in ESS round |  | 50.0% |  |  |  | 75.0% |  | 60.0% | 61.5% |
|  | Allow some | N |  | 2 |  |  |  | 1 |  | 2 | 5 |
|  |  | % in ESS round |  | 50.0% |  |  |  | 25.0% |  | 40.0% | 38.5% |
|  | Allow a few | N |  | 0 |  |  |  | 0 |  | 0 | 0 |
|  |  | % in ESS round |  | 0.0% |  |  |  | 0.0% |  | 0.0% | 0.0% |
|  | Allow none | N |  | 0 |  |  |  | 0 |  | 0 | 0 |
|  |  | % in ESS round |  | 0.0% |  |  |  | 0.0% |  | 0.0% | 0.0% |
|  |  | N |  | 4 |  |  |  | 4 |  | 5 | 13 |
|  |  | % in ESS round |  | 100.0% |  |  |  | 100.0% |  | 100.0% | 100.0% |
| Italy | Allow many to come and live here | N | 326 |  |  |  |  | 316 |  | 215 | 857 |
|  |  | % in ESS round | 21.4% |  |  |  |  | 26.3% |  | 19.5% | 22.4% |
|  | Allow some | N | 746 |  |  |  |  | 588 |  | 431 | 1765 |
|  |  | % in ESS round | 49.0% |  |  |  |  | 48.9% |  | 39.1% | 46.1% |
|  | Allow a few | N | 368 |  |  |  |  | 212 |  | 337 | 917 |
|  |  | % in ESS round | 24.2% |  |  |  |  | 17.6% |  | 30.6% | 24.0% |
|  | Allow none | N | 81 |  |  |  |  | 87 |  | 118 | 286 |
|  |  | % in ESS round | 5.3% |  |  |  |  | 7.2% |  | 10.7% | 7.5% |
|  |  | N | 1521 |  |  |  |  | 1203 |  | 1101 | 3825 |
|  |  | % in ESS round | 100.0% |  |  |  |  | 100.0% |  | 100.0% | 100.0% |
| Lithuania | Allow many to come and live here | N |  |  |  |  | 18 | 11 | 12 | 10 | 51 |
|  |  | % in ESS round |  |  |  |  | 34.6% | 26.8% | 26.7% | 23.3% | 28.2% |
|  | Allow some | N |  |  |  |  | 25 | 20 | 19 | 17 | 81 |
|  |  | % in ESS round |  |  |  |  | 48.1% | 48.8% | 42.2% | 39.5% | 44.8% |
|  | Allow a few | N |  |  |  |  | 5 | 7 | 10 | 11 | 33 |
|  |  | % in ESS round |  |  |  |  | 9.6% | 17.1% | 22.2% | 25.6% | 18.2% |
|  | Allow none | N |  |  |  |  | 4 | 3 | 4 | 5 | 16 |
|  |  | % in ESS round |  |  |  |  | 7.7% | 7.3% | 8.9% | 11.6% | 8.8% |
|  |  | N |  |  |  |  | 52 | 41 | 45 | 43 | 181 |
|  |  | % in ESS round |  |  |  |  | 100.0% | 100.0% | 100.0% | 100.0% | 100.0% |
| Luxembourg | Allow many to come and live here | N | 1 | 1 |  |  |  |  |  |  | 2 |
|  |  | % in ESS round | 33.3% | 20.0% |  |  |  |  |  |  | 25.0% |
|  | Allow some | N | 1 | 3 |  |  |  |  |  |  | 4 |
|  |  | % in ESS round | 33.3% | 60.0% |  |  |  |  |  |  | 50.0% |
|  | Allow a few | N | 1 | 1 |  |  |  |  |  |  | 2 |
|  |  | % in ESS round | 33.3% | 20.0% |  |  |  |  |  |  | 25.0% |
|  | Allow none | N | 0 | 0 |  |  |  |  |  |  | 0 |
|  |  | % in ESS round | 0.0% | 0.0% |  |  |  |  |  |  | 0.0% |
|  |  | N | 3 | 5 |  |  |  |  |  |  | 8 |
|  |  | % in ESS round | 100.0% | 100.0% |  |  |  |  |  |  | 100.0% |
| Netherlands | Allow many to come and live here | N | 31 | 38 | 54 | 41 | 34 | 51 | 46 | 65 | 360 |
|  |  | % in ESS round | 9.0% | 10.8% | 14.7% | 12.7% | 11.2% | 15.6% | 17.0% | 21.0% | 13.9% |
|  | Allow some | N | 201 | 209 | 181 | 183 | 174 | 177 | 143 | 169 | 1437 |
|  |  | % in ESS round | 58.6% | 59.2% | 49.3% | 56.8% | 57.4% | 54.3% | 52.8% | 54.5% | 55.4% |
|  | Allow a few | N | 97 | 84 | 114 | 91 | 80 | 82 | 69 | 67 | 684 |
|  |  | % in ESS round | 28.3% | 23.8% | 31.1% | 28.3% | 26.4% | 25.2% | 25.5% | 21.6% | 26.4% |
|  | Allow none | N | 14 | 22 | 18 | 7 | 15 | 16 | 13 | 9 | 114 |
|  |  | % in ESS round | 4.1% | 6.2% | 4.9% | 2.2% | 5.0% | 4.9% | 4.8% | 2.9% | 4.4% |
|  |  | N | 343 | 353 | 367 | 322 | 303 | 326 | 271 | 310 | 2595 |
|  |  | % in ESS round | 100.0% | 100.0% | 100.0% | 100.0% | 100.0% | 100.0% | 100.0% | 100.0% | 100.0% |
| Norway | Allow many to come and live here | N | 22 | 21 | 27 | 34 | 26 | 25 | 34 | 36 | 225 |
|  |  | % in ESS round | 19.5% | 18.6% | 24.3% | 28.6% | 24.3% | 24.0% | 31.2% | 33.3% | 25.5% |
|  | Allow some | N | 59 | 66 | 58 | 62 | 56 | 55 | 59 | 58 | 473 |
|  |  | % in ESS round | 52.2% | 58.4% | 52.3% | 52.1% | 52.3% | 52.9% | 54.1% | 53.7% | 53.5% |
|  | Allow a few | N | 29 | 24 | 25 | 22 | 24 | 22 | 16 | 14 | 176 |
|  |  | % in ESS round | 25.7% | 21.2% | 22.5% | 18.5% | 22.4% | 21.2% | 14.7% | 13.0% | 19.9% |
|  | Allow none | N | 3 | 2 | 1 | 1 | 1 | 2 | 0 | 0 | 10 |
|  |  | % in ESS round | 2.7% | 1.8% | .9% | .8% | .9% | 1.9% | 0.0% | 0.0% | 1.1% |
|  |  | N | 113 | 113 | 111 | 119 | 107 | 104 | 109 | 108 | 884 |
|  |  | % in ESS round | 100.0% | 100.0% | 100.0% | 100.0% | 100.0% | 100.0% | 100.0% | 100.0% | 100.0% |
| Poland | Allow many to come and live here | N | 102 | 176 | 248 | 279 | 256 | 237 | 179 | 113 | 1590 |
|  |  | % in ESS round | 15.1% | 25.2% | 33.9% | 34.1% | 32.4% | 31.6% | 21.5% | 14.9% | 26.2% |
|  | Allow some | N | 381 | 368 | 384 | 420 | 444 | 372 | 446 | 384 | 3199 |
|  |  | % in ESS round | 56.4% | 52.6% | 52.5% | 51.3% | 56.1% | 49.5% | 53.5% | 50.7% | 52.8% |
|  | Allow a few | N | 169 | 127 | 88 | 115 | 76 | 118 | 177 | 213 | 1083 |
|  |  | % in ESS round | 25.0% | 18.2% | 12.0% | 14.0% | 9.6% | 15.7% | 21.2% | 28.1% | 17.9% |
|  | Allow none | N | 24 | 28 | 12 | 5 | 15 | 24 | 32 | 47 | 187 |
|  |  | % in ESS round | 3.6% | 4.0% | 1.6% | .6% | 1.9% | 3.2% | 3.8% | 6.2% | 3.1% |
|  |  | N | 676 | 699 | 732 | 819 | 791 | 751 | 834 | 757 | 6059 |
|  |  | % in ESS round | 100.0% | 100.0% | 100.0% | 100.0% | 100.0% | 100.0% | 100.0% | 100.0% | 100.0% |
| Portugal | Allow many to come and live here | N | 14 | 8 | 12 | 12 | 9 | 12 | 19 | 39 | 125 |
|  |  | % in ESS round | 6.1% | 5.6% | 5.7% | 8.6% | 6.2% | 7.1% | 14.5% | 14.4% | 8.7% |
|  | Allow some | N | 82 | 56 | 68 | 56 | 60 | 63 | 72 | 172 | 629 |
|  |  | % in ESS round | 36.0% | 39.2% | 32.2% | 40.0% | 41.4% | 37.5% | 55.0% | 63.7% | 43.8% |
|  | Allow a few | N | 94 | 54 | 75 | 50 | 51 | 53 | 28 | 45 | 450 |
|  |  | % in ESS round | 41.2% | 37.8% | 35.5% | 35.7% | 35.2% | 31.5% | 21.4% | 16.7% | 31.3% |
|  | Allow none | N | 38 | 25 | 56 | 22 | 25 | 40 | 12 | 14 | 232 |
|  |  | % in ESS round | 16.7% | 17.5% | 26.5% | 15.7% | 17.2% | 23.8% | 9.2% | 5.2% | 16.2% |
|  |  | N | 228 | 143 | 211 | 140 | 145 | 168 | 131 | 270 | 1436 |
|  |  | % in ESS round | 100.0% | 100.0% | 100.0% | 100.0% | 100.0% | 100.0% | 100.0% | 100.0% | 100.0% |
| Russia | Allow many to come and live here | N |  |  | 742 | 645 | 826 | 827 |  | 573 | 3613 |
|  |  | % in ESS round |  |  | 44.1% | 34.0% | 38.6% | 30.4% |  | 23.3% | 33.1% |
|  | Allow some | N |  |  | 449 | 648 | 670 | 780 |  | 819 | 3366 |
|  |  | % in ESS round |  |  | 26.7% | 34.1% | 31.3% | 28.6% |  | 33.3% | 30.9% |
|  | Allow a few | N |  |  | 341 | 428 | 430 | 693 |  | 786 | 2678 |
|  |  | % in ESS round |  |  | 20.3% | 22.6% | 20.1% | 25.4% |  | 32.0% | 24.6% |
|  | Allow none | N |  |  | 151 | 177 | 214 | 424 |  | 279 | 1245 |
|  |  | % in ESS round |  |  | 9.0% | 9.3% | 10.0% | 15.6% |  | 11.4% | 11.4% |
|  |  | N |  |  | 1683 | 1898 | 2140 | 2724 |  | 2457 | 10902 |
|  |  | % in ESS round |  |  | 100.0% | 100.0% | 100.0% | 100.0% |  | 100.0% | 100.0% |
| Sweden | Allow many to come and live here | N | 67 | 70 | 75 | 64 | 69 | 61 | 82 | 61 | 549 |
|  |  | % in ESS round | 33.8% | 34.7% | 40.1% | 37.2% | 38.3% | 36.3% | 46.1% | 39.4% | 38.1% |
|  | Allow some | N | 114 | 113 | 92 | 92 | 101 | 89 | 88 | 85 | 774 |
|  |  | % in ESS round | 57.6% | 55.9% | 49.2% | 53.5% | 56.1% | 53.0% | 49.4% | 54.8% | 53.8% |
|  | Allow a few | N | 15 | 17 | 17 | 14 | 10 | 17 | 8 | 8 | 106 |
|  |  | % in ESS round | 7.6% | 8.4% | 9.1% | 8.1% | 5.6% | 10.1% | 4.5% | 5.2% | 7.4% |
|  | Allow none | N | 2 | 2 | 3 | 2 | 0 | 1 | 0 | 1 | 11 |
|  |  | % in ESS round | 1.0% | 1.0% | 1.6% | 1.2% | 0.0% | .6% | 0.0% | .6% | .8% |
|  |  | N | 198 | 202 | 187 | 172 | 180 | 168 | 178 | 155 | 1440 |
|  |  | % in ESS round | 100.0% | 100.0% | 100.0% | 100.0% | 100.0% | 100.0% | 100.0% | 100.0% | 100.0% |
| Slovenia | Allow many to come and live here | N | 4 |  | 3 | 5 | 5 | 7 | 9 | 8 | 41 |
|  |  | % in ESS round | 15.4% |  | 15.0% | 16.7% | 19.2% | 23.3% | 26.5% | 25.0% | 20.7% |
|  | Allow some | N | 15 |  | 13 | 17 | 15 | 15 | 19 | 18 | 112 |
|  |  | % in ESS round | 57.7% |  | 65.0% | 56.7% | 57.7% | 50.0% | 55.9% | 56.3% | 56.6% |
|  | Allow a few | N | 6 |  | 3 | 7 | 5 | 6 | 4 | 5 | 36 |
|  |  | % in ESS round | 23.1% |  | 15.0% | 23.3% | 19.2% | 20.0% | 11.8% | 15.6% | 18.2% |
|  | Allow none | N | 1 |  | 1 | 1 | 1 | 2 | 2 | 1 | 9 |
|  |  | % in ESS round | 3.8% |  | 5.0% | 3.3% | 3.8% | 6.7% | 5.9% | 3.1% | 4.5% |
|  |  | N | 26 |  | 20 | 30 | 26 | 30 | 34 | 32 | 198 |
|  |  | % in ESS round | 100.0% |  | 100.0% | 100.0% | 100.0% | 100.0% | 100.0% | 100.0% | 100.0% |
| Slovakia | Allow many to come and live here | N |  | 26 | 34 | 28 | 21 | 20 |  |  | 129 |
|  |  | % in ESS round |  | 34.7% | 30.6% | 25.5% | 20.8% | 16.1% |  |  | 24.8% |
|  | Allow some | N |  | 33 | 46 | 44 | 41 | 49 |  |  | 213 |
|  |  | % in ESS round |  | 44.0% | 41.4% | 40.0% | 40.6% | 39.5% |  |  | 40.9% |
|  | Allow a few | N |  | 12 | 24 | 29 | 28 | 37 |  |  | 130 |
|  |  | % in ESS round |  | 16.0% | 21.6% | 26.4% | 27.7% | 29.8% |  |  | 25.0% |
|  | Allow none | N |  | 4 | 7 | 9 | 11 | 18 |  |  | 49 |
|  |  | % in ESS round |  | 5.3% | 6.3% | 8.2% | 10.9% | 14.5% |  |  | 9.4% |
|  |  | N |  | 75 | 111 | 110 | 101 | 124 |  |  | 521 |
|  |  | % in ESS round |  | 100.0% | 100.0% | 100.0% | 100.0% | 100.0% |  |  | 100.0% |
| Turkey | Allow many to come and live here | N |  | 183 |  | 222 |  |  |  |  | 405 |
|  |  | % in ESS round |  | 17.5% |  | 17.0% |  |  |  |  | 17.2% |
|  | Allow some | N |  | 376 |  | 353 |  |  |  |  | 729 |
|  |  | % in ESS round |  | 35.9% |  | 27.0% |  |  |  |  | 30.9% |
|  | Allow a few | N |  | 195 |  | 351 |  |  |  |  | 546 |
|  |  | % in ESS round |  | 18.6% |  | 26.8% |  |  |  |  | 23.2% |
|  | Allow none | N |  | 293 |  | 383 |  |  |  |  | 676 |
|  |  | % in ESS round |  | 28.0% |  | 29.3% |  |  |  |  | 28.7% |
|  |  | N |  | 1047 |  | 1309 |  |  |  |  | 2356 |
|  |  | % in ESS round |  | 100.0% |  | 100.0% |  |  |  |  | 100.0% |
| Ukraine | Allow many to come and live here | N |  | 275 | 243 | 180 | 128 | 177 |  |  | 1003 |
|  |  | % in ESS round |  | 50.4% | 53.4% | 42.5% | 33.6% | 38.0% |  |  | 44.1% |
|  | Allow some | N |  | 159 | 108 | 157 | 154 | 149 |  |  | 727 |
|  |  | % in ESS round |  | 29.1% | 23.7% | 37.0% | 40.4% | 32.0% |  |  | 32.0% |
|  | Allow a few | N |  | 74 | 65 | 67 | 60 | 100 |  |  | 366 |
|  |  | % in ESS round |  | 13.6% | 14.3% | 15.8% | 15.7% | 21.5% |  |  | 16.1% |
|  | Allow none | N |  | 38 | 39 | 20 | 39 | 40 |  |  | 176 |
|  |  | % in ESS round |  | 7.0% | 8.6% | 4.7% | 10.2% | 8.6% |  |  | 7.7% |
|  |  | N |  | 546 | 455 | 424 | 381 | 466 |  |  | 2272 |
|  |  | % in ESS round |  | 100.0% | 100.0% | 100.0% | 100.0% | 100.0% |  |  | 100.0% |
| Total | Allow many to come and live here | N | 1637 | 1986 | 2528 | 2931 | 2799 | 3269 | 2014 | 2930 | 20094 |
|  |  | % in ESS round | 17.8% | 21.2% | 25.5% | 24.6% | 26.0% | 27.2% | 27.3% | 26.0% | 24.6% |
|  | Allow some | N | 4639 | 4359 | 4360 | 5185 | 4871 | 5271 | 3604 | 5000 | 37289 |
|  |  | % in ESS round | 50.4% | 46.6% | 44.0% | 43.5% | 45.3% | 43.8% | 48.8% | 44.4% | 45.6% |
|  | Allow a few | N | 2473 | 2209 | 2342 | 2787 | 2289 | 2562 | 1383 | 2545 | 18590 |
|  |  | % in ESS round | 26.9% | 23.6% | 23.7% | 23.4% | 21.3% | 21.3% | 18.7% | 22.6% | 22.7% |
|  | Allow none | N | 451 | 799 | 669 | 1023 | 804 | 924 | 386 | 795 | 5851 |
|  |  | % in ESS round | 4.9% | 8.5% | 6.8% | 8.6% | 7.5% | 7.7% | 5.2% | 7.1% | 7.2% |
|  |  | N | 9200 | 9353 | 9899 | 11926 | 10763 | 12026 | 7387 | 11270 | 81824 |
|  |  | % in ESS round | 100.0% | 100.0% | 100.0% | 100.0% | 100.0% | 100.0% | 100.0% | 100.0% | 100.0% |

| Table S2 Different Ethnicity **Allow Different Ethnicity** |  |  |  |  |  |  |  |  |  |  |  |
| --- | --- | --- | --- | --- | --- | --- | --- | --- | --- | --- | --- |
| Country |  |  | ESS round |  |  |  |  |  |  |  | Total |
|  |  |  | 1 | 2 | 3 | 4 | 5 | 6 | 7 | 8 |  |
| Austria | Allow many to come and live here | N | 11 | 22 | 20 |  |  |  | 25 | 20 | 98 |
|  |  | % in ESS round | 8.3% | 13.9% | 11.3% |  |  |  | 12.3% | 10.7% | 11.4% |
|  | Allow some | N | 42 | 61 | 62 |  |  |  | 70 | 58 | 293 |
|  |  | % in ESS round | 31.8% | 38.6% | 35.0% |  |  |  | 34.5% | 31.0% | 34.2% |
|  | Allow a few | N | 71 | 64 | 77 |  |  |  | 74 | 74 | 360 |
|  |  | % in ESS round | 53.8% | 40.5% | 43.5% |  |  |  | 36.5% | 39.6% | 42.0% |
|  | Allow none | N | 8 | 11 | 18 |  |  |  | 34 | 35 | 106 |
|  |  | % in ESS round | 6.1% | 7.0% | 10.2% |  |  |  | 16.7% | 18.7% | 12.4% |
|  |  | N | 132 | 158 | 177 |  |  |  | 203 | 187 | 857 |
|  |  | % in ESS round | 100.0% | 100.0% | 100.0% |  |  |  | 100.0% | 100.0% | 100.0% |
| Belgium | Allow many to come and live here | N | 14 | 23 | 24 | 29 | 24 | 18 | 19 | 41 | 192 |
|  |  | % in ESS round | 7.5% | 9.8% | 11.6% | 13.2% | 11.3% | 8.2% | 9.0% | 19.7% | 11.3% |
|  | Allow some | N | 94 | 108 | 105 | 109 | 94 | 110 | 105 | 107 | 832 |
|  |  | % in ESS round | 50.3% | 46.2% | 50.7% | 49.8% | 44.3% | 50.2% | 49.8% | 51.4% | 49.0% |
|  | Allow a few | N | 61 | 67 | 51 | 57 | 60 | 68 | 64 | 47 | 475 |
|  |  | % in ESS round | 32.6% | 28.6% | 24.6% | 26.0% | 28.3% | 31.1% | 30.3% | 22.6% | 28.0% |
|  | Allow none | N | 18 | 36 | 27 | 24 | 34 | 23 | 23 | 13 | 198 |
|  |  | % in ESS round | 9.6% | 15.4% | 13.0% | 11.0% | 16.0% | 10.5% | 10.9% | 6.3% | 11.7% |
|  |  | N | 187 | 234 | 207 | 219 | 212 | 219 | 211 | 208 | 1697 |
|  |  | % in ESS round | 100.0% | 100.0% | 100.0% | 100.0% | 100.0% | 100.0% | 100.0% | 100.0% | 100.0% |
| Bulgaria | Allow many to come and live here | N |  |  | 29 | 34 | 37 | 32 |  |  | 132 |
|  |  | % in ESS round |  |  | 33.7% | 34.3% | 32.7% | 28.3% |  |  | 32.1% |
|  | Allow some | N |  |  | 26 | 37 | 39 | 39 |  |  | 141 |
|  |  | % in ESS round |  |  | 30.2% | 37.4% | 34.5% | 34.5% |  |  | 34.3% |
|  | Allow a few | N |  |  | 18 | 18 | 23 | 26 |  |  | 85 |
|  |  | % in ESS round |  |  | 20.9% | 18.2% | 20.4% | 23.0% |  |  | 20.7% |
|  | Allow none | N |  |  | 13 | 10 | 14 | 16 |  |  | 53 |
|  |  | % in ESS round |  |  | 15.1% | 10.1% | 12.4% | 14.2% |  |  | 12.9% |
|  |  | N |  |  | 86 | 99 | 113 | 113 |  |  | 411 |
|  |  | % in ESS round |  |  | 100.0% | 100.0% | 100.0% | 100.0% |  |  | 100.0% |
| Switzerland | Allow many to come and live here | N | 14 | 15 | 18 | 16 | 19 | 15 | 15 | 18 | 130 |
|  |  | % in ESS round | 11.1% | 10.3% | 13.6% | 11.7% | 14.4% | 10.4% | 11.6% | 14.1% | 12.1% |
|  | Allow some | N | 68 | 76 | 59 | 71 | 64 | 77 | 69 | 71 | 555 |
|  |  | % in ESS round | 54.0% | 52.1% | 44.7% | 51.8% | 48.5% | 53.5% | 53.5% | 55.5% | 51.7% |
|  | Allow a few | N | 42 | 46 | 47 | 42 | 43 | 47 | 41 | 32 | 340 |
|  |  | % in ESS round | 33.3% | 31.5% | 35.6% | 30.7% | 32.6% | 32.6% | 31.8% | 25.0% | 31.7% |
|  | Allow none | N | 2 | 9 | 8 | 8 | 6 | 5 | 4 | 7 | 49 |
|  |  | % in ESS round | 1.6% | 6.2% | 6.1% | 5.8% | 4.5% | 3.5% | 3.1% | 5.5% | 4.6% |
|  |  | N | 126 | 146 | 132 | 137 | 132 | 144 | 129 | 128 | 1074 |
|  |  | % in ESS round | 100.0% | 100.0% | 100.0% | 100.0% | 100.0% | 100.0% | 100.0% | 100.0% | 100.0% |
| Cyprus | Allow many to come and live here | N |  |  | 0 | 0 | 1 | 1 |  |  | 2 |
|  |  | % in ESS round |  |  | 0.0% | 0.0% | 7.1% | 7.7% |  |  | 3.3% |
|  | Allow some | N |  |  | 2 | 2 | 2 | 1 |  |  | 7 |
|  |  | % in ESS round |  |  | 12.5% | 11.1% | 14.3% | 7.7% |  |  | 11.5% |
|  | Allow a few | N |  |  | 9 | 11 | 7 | 6 |  |  | 33 |
|  |  | % in ESS round |  |  | 56.3% | 61.1% | 50.0% | 46.2% |  |  | 54.1% |
|  | Allow none | N |  |  | 5 | 5 | 4 | 5 |  |  | 19 |
|  |  | % in ESS round |  |  | 31.3% | 27.8% | 28.6% | 38.5% |  |  | 31.1% |
|  |  | N |  |  | 16 | 18 | 14 | 13 |  |  | 61 |
|  |  | % in ESS round |  |  | 100.0% | 100.0% | 100.0% | 100.0% |  |  | 100.0% |
| Czechia | Allow many to come and live here | N | 13 | 11 |  | 11 | 16 | 7 | 4 | 5 | 67 |
|  |  | % in ESS round | 7.9% | 6.8% |  | 4.7% | 6.3% | 3.8% | 1.7% | 1.5% | 4.3% |
|  | Allow some | N | 72 | 64 |  | 81 | 74 | 49 | 77 | 78 | 495 |
|  |  | % in ESS round | 43.6% | 39.5% |  | 34.5% | 28.9% | 26.8% | 32.1% | 23.8% | 31.5% |
|  | Allow a few | N | 62 | 60 |  | 98 | 112 | 82 | 97 | 136 | 647 |
|  |  | % in ESS round | 37.6% | 37.0% |  | 41.7% | 43.8% | 44.8% | 40.4% | 41.5% | 41.2% |
|  | Allow none | N | 18 | 27 |  | 45 | 54 | 45 | 62 | 109 | 360 |
|  |  | % in ESS round | 10.9% | 16.7% |  | 19.1% | 21.1% | 24.6% | 25.8% | 33.2% | 22.9% |
|  |  | N | 165 | 162 |  | 235 | 256 | 183 | 240 | 328 | 1569 |
|  |  | % in ESS round | 100.0% | 100.0% |  | 100.0% | 100.0% | 100.0% | 100.0% | 100.0% | 100.0% |
| Germany | Allow many to come and live here | N | 238 | 211 | 192 | 389 | 295 | 378 | 566 | 488 | 2757 |
|  |  | % in ESS round | 12.3% | 11.3% | 10.2% | 20.0% | 16.2% | 21.2% | 30.9% | 26.1% | 18.5% |
|  | Allow some | N | 930 | 806 | 820 | 856 | 879 | 908 | 933 | 916 | 7048 |
|  |  | % in ESS round | 48.2% | 43.3% | 43.5% | 44.1% | 48.1% | 50.8% | 50.9% | 49.0% | 47.2% |
|  | Allow a few | N | 639 | 631 | 652 | 554 | 515 | 437 | 272 | 437 | 4137 |
|  |  | % in ESS round | 33.1% | 33.9% | 34.6% | 28.5% | 28.2% | 24.5% | 14.8% | 23.4% | 27.7% |
|  | Allow none | N | 123 | 213 | 223 | 144 | 137 | 64 | 63 | 29 | 996 |
|  |  | % in ESS round | 6.4% | 11.4% | 11.8% | 7.4% | 7.5% | 3.6% | 3.4% | 1.6% | 6.7% |
|  |  | N | 1930 | 1861 | 1887 | 1943 | 1826 | 1787 | 1834 | 1870 | 14938 |
|  |  | % in ESS round | 100.0% | 100.0% | 100.0% | 100.0% | 100.0% | 100.0% | 100.0% | 100.0% | 100.0% |
| Denmark | Allow many to come and live here | N | 16 | 19 | 13 | 23 | 22 | 23 | 20 |  | 136 |
|  |  | % in ESS round | 11.7% | 13.9% | 10.9% | 18.5% | 18.6% | 17.8% | 15.4% |  | 15.2% |
|  | Allow some | N | 55 | 54 | 55 | 57 | 55 | 61 | 59 |  | 396 |
|  |  | % in ESS round | 40.1% | 39.4% | 46.2% | 46.0% | 46.6% | 47.3% | 45.4% |  | 44.3% |
|  | Allow a few | N | 58 | 55 | 45 | 41 | 36 | 41 | 44 |  | 320 |
|  |  | % in ESS round | 42.3% | 40.1% | 37.8% | 33.1% | 30.5% | 31.8% | 33.8% |  | 35.8% |
|  | Allow none | N | 8 | 9 | 6 | 3 | 5 | 4 | 7 |  | 42 |
|  |  | % in ESS round | 5.8% | 6.6% | 5.0% | 2.4% | 4.2% | 3.1% | 5.4% |  | 4.7% |
|  |  | N | 137 | 137 | 119 | 124 | 118 | 129 | 130 |  | 894 |
|  |  | % in ESS round | 100.0% | 100.0% | 100.0% | 100.0% | 100.0% | 100.0% | 100.0% |  | 100.0% |
| Estonia | Allow many to come and live here | N |  | 2 | 2 | 2 | 2 | 3 | 2 | 2 | 15 |
|  |  | % in ESS round |  | 11.8% | 12.5% | 10.0% | 10.0% | 15.0% | 12.5% | 9.1% | 11.5% |
|  | Allow some | N |  | 5 | 6 | 8 | 9 | 8 | 8 | 9 | 53 |
|  |  | % in ESS round |  | 29.4% | 37.5% | 40.0% | 45.0% | 40.0% | 50.0% | 40.9% | 40.5% |
|  | Allow a few | N |  | 7 | 5 | 7 | 7 | 7 | 5 | 9 | 47 |
|  |  | % in ESS round |  | 41.2% | 31.3% | 35.0% | 35.0% | 35.0% | 31.3% | 40.9% | 35.9% |
|  | Allow none | N |  | 3 | 3 | 3 | 2 | 2 | 1 | 2 | 16 |
|  |  | % in ESS round |  | 17.6% | 18.8% | 15.0% | 10.0% | 10.0% | 6.3% | 9.1% | 12.2% |
|  |  | N |  | 17 | 16 | 20 | 20 | 20 | 16 | 22 | 131 |
|  |  | % in ESS round |  | 100.0% | 100.0% | 100.0% | 100.0% | 100.0% | 100.0% | 100.0% | 100.0% |
| Spain | Allow many to come and live here | N | 137 | 178 | 143 | 102 | 200 | 245 | 258 | 304 | 1567 |
|  |  | % in ESS round | 17.9% | 19.3% | 14.7% | 11.4% | 18.2% | 25.5% | 27.6% | 36.3% | 21.2% |
|  | Allow some | N | 266 | 327 | 376 | 327 | 403 | 349 | 352 | 282 | 2682 |
|  |  | % in ESS round | 34.7% | 35.4% | 38.8% | 36.4% | 36.7% | 36.3% | 37.7% | 33.7% | 36.3% |
|  | Allow a few | N | 311 | 336 | 340 | 355 | 373 | 286 | 242 | 201 | 2444 |
|  |  | % in ESS round | 40.5% | 36.4% | 35.1% | 39.5% | 33.9% | 29.7% | 25.9% | 24.0% | 33.1% |
|  | Allow none | N | 53 | 82 | 111 | 114 | 123 | 82 | 82 | 50 | 697 |
|  |  | % in ESS round | 6.9% | 8.9% | 11.4% | 12.7% | 11.2% | 8.5% | 8.8% | 6.0% | 9.4% |
|  |  | N | 767 | 923 | 970 | 898 | 1099 | 962 | 934 | 837 | 7390 |
|  |  | % in ESS round | 100.0% | 100.0% | 100.0% | 100.0% | 100.0% | 100.0% | 100.0% | 100.0% | 100.0% |
| Finland | Allow many to come and live here | N | 12 | 15 | 13 | 17 | 10 | 17 | 20 | 21 | 125 |
|  |  | % in ESS round | 8.2% | 10.9% | 8.4% | 11.7% | 7.5% | 12.6% | 16.3% | 15.6% | 11.3% |
|  | Allow some | N | 48 | 46 | 58 | 58 | 50 | 53 | 48 | 56 | 417 |
|  |  | % in ESS round | 32.7% | 33.3% | 37.7% | 40.0% | 37.3% | 39.3% | 39.0% | 41.5% | 37.5% |
|  | Allow a few | N | 75 | 67 | 73 | 63 | 67 | 57 | 48 | 49 | 499 |
|  |  | % in ESS round | 51.0% | 48.6% | 47.4% | 43.4% | 50.0% | 42.2% | 39.0% | 36.3% | 44.9% |
|  | Allow none | N | 12 | 10 | 10 | 7 | 7 | 8 | 7 | 9 | 70 |
|  |  | % in ESS round | 8.2% | 7.2% | 6.5% | 4.8% | 5.2% | 5.9% | 5.7% | 6.7% | 6.3% |
|  |  | N | 147 | 138 | 154 | 145 | 134 | 135 | 123 | 135 | 1111 |
|  |  | % in ESS round | 100.0% | 100.0% | 100.0% | 100.0% | 100.0% | 100.0% | 100.0% | 100.0% | 100.0% |
| France | Allow many to come and live here | N | 93 | 119 | 97 | 103 | 124 | 126 | 102 | 180 | 944 |
|  |  | % in ESS round | 9.3% | 11.4% | 9.2% | 9.6% | 11.8% | 13.0% | 13.8% | 18.1% | 11.9% |
|  | Allow some | N | 492 | 511 | 502 | 557 | 498 | 467 | 372 | 488 | 3887 |
|  |  | % in ESS round | 49.4% | 48.9% | 47.4% | 51.9% | 47.2% | 48.2% | 50.4% | 48.9% | 49.0% |
|  | Allow a few | N | 308 | 300 | 373 | 350 | 341 | 289 | 208 | 207 | 2376 |
|  |  | % in ESS round | 31.0% | 28.7% | 35.3% | 32.6% | 32.3% | 29.9% | 28.2% | 20.8% | 30.0% |
|  | Allow none | N | 102 | 114 | 86 | 64 | 92 | 86 | 56 | 122 | 722 |
|  |  | % in ESS round | 10.3% | 10.9% | 8.1% | 6.0% | 8.7% | 8.9% | 7.6% | 12.2% | 9.1% |
|  |  | N | 995 | 1044 | 1058 | 1074 | 1055 | 968 | 738 | 997 | 7929 |
|  |  | % in ESS round | 100.0% | 100.0% | 100.0% | 100.0% | 100.0% | 100.0% | 100.0% | 100.0% | 100.0% |
| United Kingdom | Allow many to come and live here | N | 117 | 76 | 88 | 94 | 89 | 92 | 114 | 185 | 855 |
|  |  | % in ESS round | 10.4% | 8.3% | 9.1% | 8.6% | 9.3% | 10.3% | 13.0% | 18.8% | 10.9% |
|  | Allow some | N | 501 | 384 | 456 | 569 | 428 | 431 | 421 | 499 | 3689 |
|  |  | % in ESS round | 44.5% | 41.7% | 47.4% | 52.0% | 44.9% | 48.2% | 47.9% | 50.7% | 47.2% |
|  | Allow a few | N | 365 | 352 | 327 | 297 | 296 | 270 | 238 | 248 | 2393 |
|  |  | % in ESS round | 32.4% | 38.2% | 34.0% | 27.1% | 31.0% | 30.2% | 27.1% | 25.2% | 30.6% |
|  | Allow none | N | 144 | 109 | 91 | 134 | 141 | 101 | 105 | 53 | 878 |
|  |  | % in ESS round | 12.8% | 11.8% | 9.5% | 12.2% | 14.8% | 11.3% | 12.0% | 5.4% | 11.2% |
|  |  | N | 1127 | 921 | 962 | 1094 | 954 | 894 | 878 | 985 | 7815 |
|  |  | % in ESS round | 100.0% | 100.0% | 100.0% | 100.0% | 100.0% | 100.0% | 100.0% | 100.0% | 100.0% |
| Greece | Allow many to come and live here | N | 6 | 9 |  | 10 | 9 |  |  |  | 34 |
|  |  | % in ESS round | 4.1% | 4.8% |  | 4.7% | 4.9% |  |  |  | 4.7% |
|  | Allow some | N | 16 | 30 |  | 29 | 24 |  |  |  | 99 |
|  |  | % in ESS round | 11.0% | 16.0% |  | 13.6% | 13.2% |  |  |  | 13.6% |
|  | Allow a few | N | 99 | 110 |  | 119 | 86 |  |  |  | 414 |
|  |  | % in ESS round | 67.8% | 58.8% |  | 55.6% | 47.3% |  |  |  | 56.8% |
|  | Allow none | N | 25 | 38 |  | 56 | 63 |  |  |  | 182 |
|  |  | % in ESS round | 17.1% | 20.3% |  | 26.2% | 34.6% |  |  |  | 25.0% |
|  |  | N | 146 | 187 |  | 214 | 182 |  |  |  | 729 |
|  |  | % in ESS round | 100.0% | 100.0% |  | 100.0% | 100.0% |  |  |  | 100.0% |
| Croatia | Allow many to come and live here | N |  |  |  | 17 | 16 |  |  |  | 33 |
|  |  | % in ESS round |  |  |  | 27.9% | 27.1% |  |  |  | 27.5% |
|  | Allow some | N |  |  |  | 26 | 21 |  |  |  | 47 |
|  |  | % in ESS round |  |  |  | 42.6% | 35.6% |  |  |  | 39.2% |
|  | Allow a few | N |  |  |  | 14 | 16 |  |  |  | 30 |
|  |  | % in ESS round |  |  |  | 23.0% | 27.1% |  |  |  | 25.0% |
|  | Allow none | N |  |  |  | 4 | 6 |  |  |  | 10 |
|  |  | % in ESS round |  |  |  | 6.6% | 10.2% |  |  |  | 8.3% |
|  |  | N |  |  |  | 61 | 59 |  |  |  | 120 |
|  |  | % in ESS round |  |  |  | 100.0% | 100.0% |  |  |  | 100.0% |
| Hungary | Allow many to come and live here | N | 4 | 8 | 7 | 8 | 17 | 10 | 9 | 5 | 68 |
|  |  | % in ESS round | 2.6% | 4.0% | 4.0% | 3.8% | 7.3% | 4.6% | 3.8% | 2.2% | 4.1% |
|  | Allow some | N | 22 | 36 | 30 | 30 | 40 | 46 | 40 | 24 | 268 |
|  |  | % in ESS round | 14.2% | 18.2% | 16.9% | 14.4% | 17.1% | 21.1% | 16.8% | 10.7% | 16.2% |
|  | Allow a few | N | 101 | 110 | 87 | 112 | 115 | 109 | 109 | 80 | 823 |
|  |  | % in ESS round | 65.2% | 55.6% | 49.2% | 53.6% | 49.1% | 50.0% | 45.8% | 35.7% | 49.8% |
|  | Allow none | N | 28 | 44 | 53 | 59 | 62 | 53 | 80 | 115 | 494 |
|  |  | % in ESS round | 18.1% | 22.2% | 29.9% | 28.2% | 26.5% | 24.3% | 33.6% | 51.3% | 29.9% |
|  |  | N | 155 | 198 | 177 | 209 | 234 | 218 | 238 | 224 | 1653 |
|  |  | % in ESS round | 100.0% | 100.0% | 100.0% | 100.0% | 100.0% | 100.0% | 100.0% | 100.0% | 100.0% |
| Ireland | Allow many to come and live here | N | 9 | 13 | 9 | 10 | 8 | 14 | 10 | 10 | 83 |
|  |  | % in ESS round | 14.5% | 18.6% | 15.8% | 13.9% | 9.8% | 17.3% | 14.9% | 14.7% | 14.8% |
|  | Allow some | N | 33 | 35 | 33 | 35 | 38 | 36 | 24 | 28 | 262 |
|  |  | % in ESS round | 53.2% | 50.0% | 57.9% | 48.6% | 46.3% | 44.4% | 35.8% | 41.2% | 46.9% |
|  | Allow a few | N | 18 | 16 | 11 | 19 | 22 | 21 | 26 | 23 | 156 |
|  |  | % in ESS round | 29.0% | 22.9% | 19.3% | 26.4% | 26.8% | 25.9% | 38.8% | 33.8% | 27.9% |
|  | Allow none | N | 2 | 6 | 4 | 8 | 14 | 10 | 7 | 7 | 58 |
|  |  | % in ESS round | 3.2% | 8.6% | 7.0% | 11.1% | 17.1% | 12.3% | 10.4% | 10.3% | 10.4% |
|  |  | N | 62 | 70 | 57 | 72 | 82 | 81 | 67 | 68 | 559 |
|  |  | % in ESS round | 100.0% | 100.0% | 100.0% | 100.0% | 100.0% | 100.0% | 100.0% | 100.0% | 100.0% |
| Israel | Allow many to come and live here | N | 1 |  |  | 3 | 3 | 2 | 5 | 3 | 17 |
|  |  | % in ESS round | 6.3% |  |  | 11.1% | 8.8% | 3.9% | 9.3% | 7.0% | 7.6% |
|  | Allow some | N | 5 |  |  | 7 | 10 | 10 | 17 | 14 | 63 |
|  |  | % in ESS round | 31.3% |  |  | 25.9% | 29.4% | 19.6% | 31.5% | 32.6% | 28.0% |
|  | Allow a few | N | 7 |  |  | 9 | 11 | 16 | 20 | 14 | 77 |
|  |  | % in ESS round | 43.8% |  |  | 33.3% | 32.4% | 31.4% | 37.0% | 32.6% | 34.2% |
|  | Allow none | N | 3 |  |  | 8 | 10 | 23 | 12 | 12 | 68 |
|  |  | % in ESS round | 18.8% |  |  | 29.6% | 29.4% | 45.1% | 22.2% | 27.9% | 30.2% |
|  |  | N | 16 |  |  | 27 | 34 | 51 | 54 | 43 | 225 |
|  |  | % in ESS round | 100.0% |  |  | 100.0% | 100.0% | 100.0% | 100.0% | 100.0% | 100.0% |
| Iceland | Allow many to come and live here | N |  | 1 |  |  |  | 2 |  | 2 | 5 |
|  |  | % in ESS round |  | 25.0% |  |  |  | 40.0% |  | 50.0% | 38.5% |
|  | Allow some | N |  | 2 |  |  |  | 2 |  | 2 | 6 |
|  |  | % in ESS round |  | 50.0% |  |  |  | 40.0% |  | 50.0% | 46.2% |
|  | Allow a few | N |  | 1 |  |  |  | 1 |  | 0 | 2 |
|  |  | % in ESS round |  | 25.0% |  |  |  | 20.0% |  | 0.0% | 15.4% |
|  | Allow none | N |  | 0 |  |  |  | 0 |  | 0 | 0 |
|  |  | % in ESS round |  | 0.0% |  |  |  | 0.0% |  | 0.0% | 0.0% |
|  |  | N |  | 4 |  |  |  | 5 |  | 4 | 13 |
|  |  | % in ESS round |  | 100.0% |  |  |  | 100.0% |  | 100.0% | 100.0% |
| Italy | Allow many to come and live here | N | 243 |  |  |  |  | 256 |  | 139 | 638 |
|  |  | % in ESS round | 16.0% |  |  |  |  | 21.2% |  | 12.6% | 16.6% |
|  | Allow some | N | 727 |  |  |  |  | 567 |  | 351 | 1645 |
|  |  | % in ESS round | 47.7% |  |  |  |  | 46.9% |  | 31.7% | 42.8% |
|  | Allow a few | N | 444 |  |  |  |  | 245 |  | 415 | 1104 |
|  |  | % in ESS round | 29.2% |  |  |  |  | 20.2% |  | 37.5% | 28.8% |
|  | Allow none | N | 109 |  |  |  |  | 142 |  | 202 | 453 |
|  |  | % in ESS round | 7.2% |  |  |  |  | 11.7% |  | 18.2% | 11.8% |
|  |  | N | 1523 |  |  |  |  | 1210 |  | 1107 | 3840 |
|  |  | % in ESS round | 100.0% |  |  |  |  | 100.0% |  | 100.0% | 100.0% |
| Lithuania | Allow many to come and live here | N |  |  |  |  | 14 | 7 | 6 | 3 | 30 |
|  |  | % in ESS round |  |  |  |  | 26.4% | 17.5% | 13.0% | 7.0% | 16.5% |
|  | Allow some | N |  |  |  |  | 25 | 20 | 19 | 18 | 82 |
|  |  | % in ESS round |  |  |  |  | 47.2% | 50.0% | 41.3% | 41.9% | 45.1% |
|  | Allow a few | N |  |  |  |  | 10 | 9 | 15 | 13 | 47 |
|  |  | % in ESS round |  |  |  |  | 18.9% | 22.5% | 32.6% | 30.2% | 25.8% |
|  | Allow none | N |  |  |  |  | 4 | 4 | 6 | 9 | 23 |
|  |  | % in ESS round |  |  |  |  | 7.5% | 10.0% | 13.0% | 20.9% | 12.6% |
|  |  | N |  |  |  |  | 53 | 40 | 46 | 43 | 182 |
|  |  | % in ESS round |  |  |  |  | 100.0% | 100.0% | 100.0% | 100.0% | 100.0% |
| Luxembourg | Allow many to come and live here | N | 0 | 1 |  |  |  |  |  |  | 1 |
|  |  | % in ESS round | 0.0% | 16.7% |  |  |  |  |  |  | 12.5% |
|  | Allow some | N | 1 | 2 |  |  |  |  |  |  | 3 |
|  |  | % in ESS round | 50.0% | 33.3% |  |  |  |  |  |  | 37.5% |
|  | Allow a few | N | 1 | 2 |  |  |  |  |  |  | 3 |
|  |  | % in ESS round | 50.0% | 33.3% |  |  |  |  |  |  | 37.5% |
|  | Allow none | N | 0 | 1 |  |  |  |  |  |  | 1 |
|  |  | % in ESS round | 0.0% | 16.7% |  |  |  |  |  |  | 12.5% |
|  |  | N | 2 | 6 |  |  |  |  |  |  | 8 |
|  |  | % in ESS round | 100.0% | 100.0% |  |  |  |  |  |  | 100.0% |
| Netherlands | Allow many to come and live here | N | 25 | 22 | 34 | 36 | 37 | 46 | 43 | 59 | 302 |
|  |  | % in ESS round | 7.3% | 6.3% | 9.2% | 11.2% | 12.2% | 14.1% | 15.9% | 19.0% | 11.6% |
|  | Allow some | N | 191 | 184 | 167 | 172 | 154 | 164 | 132 | 162 | 1326 |
|  |  | % in ESS round | 55.8% | 52.4% | 45.4% | 53.4% | 50.7% | 50.2% | 48.7% | 52.3% | 51.1% |
|  | Allow a few | N | 104 | 108 | 137 | 99 | 91 | 100 | 78 | 75 | 792 |
|  |  | % in ESS round | 30.4% | 30.8% | 37.2% | 30.7% | 29.9% | 30.6% | 28.8% | 24.2% | 30.5% |
|  | Allow none | N | 22 | 37 | 30 | 15 | 22 | 17 | 18 | 14 | 175 |
|  |  | % in ESS round | 6.4% | 10.5% | 8.2% | 4.7% | 7.2% | 5.2% | 6.6% | 4.5% | 6.7% |
|  |  | N | 342 | 351 | 368 | 322 | 304 | 327 | 271 | 310 | 2595 |
|  |  | % in ESS round | 100.0% | 100.0% | 100.0% | 100.0% | 100.0% | 100.0% | 100.0% | 100.0% | 100.0% |
| Norway | Allow many to come and live here | N | 13 | 11 | 17 | 19 | 19 | 20 | 24 | 30 | 153 |
|  |  | % in ESS round | 11.5% | 9.8% | 15.3% | 16.1% | 17.8% | 19.2% | 21.8% | 28.0% | 17.3% |
|  | Allow some | N | 52 | 54 | 50 | 59 | 53 | 54 | 62 | 54 | 438 |
|  |  | % in ESS round | 46.0% | 48.2% | 45.0% | 50.0% | 49.5% | 51.9% | 56.4% | 50.5% | 49.7% |
|  | Allow a few | N | 43 | 41 | 41 | 36 | 32 | 27 | 24 | 22 | 266 |
|  |  | % in ESS round | 38.1% | 36.6% | 36.9% | 30.5% | 29.9% | 26.0% | 21.8% | 20.6% | 30.2% |
|  | Allow none | N | 5 | 6 | 3 | 4 | 3 | 3 | 0 | 1 | 25 |
|  |  | % in ESS round | 4.4% | 5.4% | 2.7% | 3.4% | 2.8% | 2.9% | 0.0% | .9% | 2.8% |
|  |  | N | 113 | 112 | 111 | 118 | 107 | 104 | 110 | 107 | 882 |
|  |  | % in ESS round | 100.0% | 100.0% | 100.0% | 100.0% | 100.0% | 100.0% | 100.0% | 100.0% | 100.0% |
| Poland | Allow many to come and live here | N | 90 | 140 | 208 | 214 | 188 | 190 | 120 | 47 | 1197 |
|  |  | % in ESS round | 13.3% | 20.1% | 28.3% | 26.1% | 23.8% | 25.2% | 14.5% | 6.2% | 19.7% |
|  | Allow some | N | 343 | 372 | 365 | 431 | 431 | 388 | 400 | 300 | 3030 |
|  |  | % in ESS round | 50.7% | 53.3% | 49.6% | 52.6% | 54.6% | 51.4% | 48.2% | 39.6% | 50.0% |
|  | Allow a few | N | 198 | 140 | 128 | 148 | 151 | 131 | 245 | 321 | 1462 |
|  |  | % in ESS round | 29.2% | 20.1% | 17.4% | 18.1% | 19.1% | 17.4% | 29.5% | 42.4% | 24.1% |
|  | Allow none | N | 46 | 46 | 35 | 26 | 19 | 46 | 65 | 89 | 372 |
|  |  | % in ESS round | 6.8% | 6.6% | 4.8% | 3.2% | 2.4% | 6.1% | 7.8% | 11.8% | 6.1% |
|  |  | N | 677 | 698 | 736 | 819 | 789 | 755 | 830 | 757 | 6061 |
|  |  | % in ESS round | 100.0% | 100.0% | 100.0% | 100.0% | 100.0% | 100.0% | 100.0% | 100.0% | 100.0% |
| Portugal | Allow many to come and live here | N | 10 | 5 | 9 | 10 | 7 | 10 | 14 | 27 | 92 |
|  |  | % in ESS round | 4.3% | 3.5% | 4.3% | 7.1% | 4.8% | 5.9% | 10.7% | 10.0% | 6.4% |
|  | Allow some | N | 70 | 52 | 64 | 53 | 55 | 54 | 55 | 153 | 556 |
|  |  | % in ESS round | 30.4% | 36.1% | 30.3% | 37.9% | 37.9% | 32.0% | 42.0% | 56.5% | 38.6% |
|  | Allow a few | N | 97 | 54 | 75 | 54 | 55 | 59 | 46 | 67 | 507 |
|  |  | % in ESS round | 42.2% | 37.5% | 35.5% | 38.6% | 37.9% | 34.9% | 35.1% | 24.7% | 35.2% |
|  | Allow none | N | 53 | 33 | 63 | 23 | 28 | 46 | 16 | 24 | 286 |
|  |  | % in ESS round | 23.0% | 22.9% | 29.9% | 16.4% | 19.3% | 27.2% | 12.2% | 8.9% | 19.8% |
|  |  | N | 230 | 144 | 211 | 140 | 145 | 169 | 131 | 271 | 1441 |
|  |  | % in ESS round | 100.0% | 100.0% | 100.0% | 100.0% | 100.0% | 100.0% | 100.0% | 100.0% | 100.0% |
| Russia | Allow many to come and live here | N |  |  | 164 | 200 | 300 | 259 |  | 249 | 1172 |
|  |  | % in ESS round |  |  | 9.8% | 10.6% | 13.9% | 9.5% |  | 10.0% | 10.7% |
|  | Allow some | N |  |  | 518 | 538 | 657 | 769 |  | 642 | 3124 |
|  |  | % in ESS round |  |  | 31.1% | 28.5% | 30.4% | 28.1% |  | 25.8% | 28.5% |
|  | Allow a few | N |  |  | 538 | 682 | 834 | 967 |  | 1145 | 4166 |
|  |  | % in ESS round |  |  | 32.3% | 36.1% | 38.6% | 35.4% |  | 46.0% | 38.1% |
|  | Allow none | N |  |  | 448 | 470 | 372 | 737 |  | 454 | 2481 |
|  |  | % in ESS round |  |  | 26.9% | 24.9% | 17.2% | 27.0% |  | 18.2% | 22.7% |
|  |  | N |  |  | 1668 | 1890 | 2163 | 2732 |  | 2490 | 10943 |
|  |  | % in ESS round |  |  | 100.0% | 100.0% | 100.0% | 100.0% |  | 100.0% | 100.0% |
| Sweden | Allow many to come and live here | N | 61 | 64 | 66 | 60 | 66 | 60 | 80 | 57 | 514 |
|  |  | % in ESS round | 31.0% | 31.7% | 35.1% | 35.1% | 36.7% | 35.9% | 44.9% | 36.5% | 35.7% |
|  | Allow some | N | 111 | 110 | 96 | 89 | 99 | 86 | 89 | 87 | 767 |
|  |  | % in ESS round | 56.3% | 54.5% | 51.1% | 52.0% | 55.0% | 51.5% | 50.0% | 55.8% | 53.3% |
|  | Allow a few | N | 22 | 25 | 23 | 20 | 15 | 19 | 9 | 11 | 144 |
|  |  | % in ESS round | 11.2% | 12.4% | 12.2% | 11.7% | 8.3% | 11.4% | 5.1% | 7.1% | 10.0% |
|  | Allow none | N | 3 | 3 | 3 | 2 | 0 | 2 | 0 | 1 | 14 |
|  |  | % in ESS round | 1.5% | 1.5% | 1.6% | 1.2% | 0.0% | 1.2% | 0.0% | .6% | 1.0% |
|  |  | N | 197 | 202 | 188 | 171 | 180 | 167 | 178 | 156 | 1439 |
|  |  | % in ESS round | 100.0% | 100.0% | 100.0% | 100.0% | 100.0% | 100.0% | 100.0% | 100.0% | 100.0% |
| Slovenia | Allow many to come and live here | N | 2 |  | 2 | 4 | 4 | 5 | 7 | 3 | 27 |
|  |  | % in ESS round | 8.0% |  | 10.0% | 13.3% | 14.8% | 16.7% | 20.6% | 9.4% | 13.6% |
|  | Allow some | N | 13 |  | 12 | 16 | 14 | 15 | 17 | 16 | 103 |
|  |  | % in ESS round | 52.0% |  | 60.0% | 53.3% | 51.9% | 50.0% | 50.0% | 50.0% | 52.0% |
|  | Allow a few | N | 8 |  | 5 | 8 | 7 | 8 | 7 | 11 | 54 |
|  |  | % in ESS round | 32.0% |  | 25.0% | 26.7% | 25.9% | 26.7% | 20.6% | 34.4% | 27.3% |
|  | Allow none | N | 2 |  | 1 | 2 | 2 | 2 | 3 | 2 | 14 |
|  |  | % in ESS round | 8.0% |  | 5.0% | 6.7% | 7.4% | 6.7% | 8.8% | 6.3% | 7.1% |
|  |  | N | 25 |  | 20 | 30 | 27 | 30 | 34 | 32 | 198 |
|  |  | % in ESS round | 100.0% |  | 100.0% | 100.0% | 100.0% | 100.0% | 100.0% | 100.0% | 100.0% |
| Slovakia | Allow many to come and live here | N |  | 16 | 23 | 21 | 17 | 12 |  |  | 89 |
|  |  | % in ESS round |  | 21.9% | 20.4% | 19.1% | 17.0% | 9.7% |  |  | 17.1% |
|  | Allow some | N |  | 35 | 50 | 41 | 36 | 39 |  |  | 201 |
|  |  | % in ESS round |  | 47.9% | 44.2% | 37.3% | 36.0% | 31.5% |  |  | 38.7% |
|  | Allow a few | N |  | 14 | 29 | 35 | 32 | 48 |  |  | 158 |
|  |  | % in ESS round |  | 19.2% | 25.7% | 31.8% | 32.0% | 38.7% |  |  | 30.4% |
|  | Allow none | N |  | 8 | 11 | 13 | 15 | 25 |  |  | 72 |
|  |  | % in ESS round |  | 11.0% | 9.7% | 11.8% | 15.0% | 20.2% |  |  | 13.8% |
|  |  | N |  | 73 | 113 | 110 | 100 | 124 |  |  | 520 |
|  |  | % in ESS round |  | 100.0% | 100.0% | 100.0% | 100.0% | 100.0% |  |  | 100.0% |
| Turkey | Allow many to come and live here | N |  | 99 |  | 135 |  |  |  |  | 234 |
|  |  | % in ESS round |  | 9.5% |  | 10.2% |  |  |  |  | 9.9% |
|  | Allow some | N |  | 245 |  | 348 |  |  |  |  | 593 |
|  |  | % in ESS round |  | 23.5% |  | 26.4% |  |  |  |  | 25.1% |
|  | Allow a few | N |  | 217 |  | 388 |  |  |  |  | 605 |
|  |  | % in ESS round |  | 20.8% |  | 29.4% |  |  |  |  | 25.6% |
|  | Allow none | N |  | 480 |  | 447 |  |  |  |  | 927 |
|  |  | % in ESS round |  | 46.1% |  | 33.9% |  |  |  |  | 39.3% |
|  |  | N |  | 1041 |  | 1318 |  |  |  |  | 2359 |
|  |  | % in ESS round |  | 100.0% |  | 100.0% |  |  |  |  | 100.0% |
| Ukraine | Allow many to come and live here | N |  | 156 | 144 | 93 | 62 | 77 |  |  | 532 |
|  |  | % in ESS round |  | 28.8% | 32.4% | 21.5% | 16.4% | 16.9% |  |  | 23.6% |
|  | Allow some | N |  | 194 | 111 | 155 | 143 | 140 |  |  | 743 |
|  |  | % in ESS round |  | 35.8% | 24.9% | 35.9% | 37.7% | 30.7% |  |  | 33.0% |
|  | Allow a few | N |  | 135 | 120 | 143 | 90 | 148 |  |  | 636 |
|  |  | % in ESS round |  | 24.9% | 27.0% | 33.1% | 23.7% | 32.5% |  |  | 28.2% |
|  | Allow none | N |  | 57 | 70 | 41 | 84 | 91 |  |  | 343 |
|  |  | % in ESS round |  | 10.5% | 15.7% | 9.5% | 22.2% | 20.0% |  |  | 15.2% |
|  |  | N |  | 542 | 445 | 432 | 379 | 456 |  |  | 2254 |
|  |  | % in ESS round |  | 100.0% | 100.0% | 100.0% | 100.0% | 100.0% |  |  | 100.0% |
| Total | Allow many to come and live here | N | 1129 | 1236 | 1322 | 1660 | 1606 | 1927 | 1463 | 1898 | 12241 |
|  |  | % in ESS round | 12.3% | 13.2% | 13.4% | 13.9% | 14.9% | 16.0% | 19.8% | 16.8% | 14.9% |
|  | Allow some | N | 4152 | 3793 | 4023 | 4761 | 4395 | 4943 | 3369 | 4415 | 33851 |
|  |  | % in ESS round | 45.1% | 40.5% | 40.7% | 39.9% | 40.8% | 41.1% | 45.6% | 39.0% | 41.3% |
|  | Allow a few | N | 3134 | 2958 | 3211 | 3779 | 3447 | 3524 | 1912 | 3637 | 25602 |
|  |  | % in ESS round | 34.1% | 31.6% | 32.5% | 31.7% | 32.0% | 29.3% | 25.9% | 32.2% | 31.3% |
|  | Allow none | N | 786 | 1382 | 1322 | 1739 | 1323 | 1642 | 651 | 1359 | 10204 |
|  |  | % in ESS round | 8.5% | 14.8% | 13.4% | 14.6% | 12.3% | 13.6% | 8.8% | 12.0% | 12.5% |
|  |  | N | 9201 | 9369 | 9878 | 11939 | 10771 | 12036 | 7395 | 11309 | 81898 |
|  |  | % in ESS round | 100.0% | 100.0% | 100.0% | 100.0% | 100.0% | 100.0% | 100.0% | 100.0% | 100.0% |

Table S3) Poorer Countries

| **Poorer Countries** |  |  |  |  |  |  |  |  |  |  |  |
| --- | --- | --- | --- | --- | --- | --- | --- | --- | --- | --- | --- |
| Country |  |  | ESS round |  |  |  |  |  |  |  | Total |
|  |  |  | 1 | 2 | 3 | 4 | 5 | 6 | 7 | 8 |  |
| Austria | Allow many to come and live here | N | 12 | 22 | 20 |  |  |  | 25 | 21 | 100 |
|  |  | % in ESS round | 9.3% | 14.0% | 11.4% |  |  |  | 12.6% | 11.2% | 11.8% |
|  | Allow some | N | 45 | 64 | 66 |  |  |  | 62 | 50 | 287 |
|  |  | % in ESS round | 34.9% | 40.8% | 37.5% |  |  |  | 31.2% | 26.6% | 33.8% |
|  | Allow a few | N | 63 | 56 | 70 |  |  |  | 69 | 72 | 330 |
|  |  | % in ESS round | 48.8% | 35.7% | 39.8% |  |  |  | 34.7% | 38.3% | 38.9% |
|  | Allow none | N | 9 | 15 | 20 |  |  |  | 43 | 45 | 132 |
|  |  | % in ESS round | 7.0% | 9.6% | 11.4% |  |  |  | 21.6% | 23.9% | 15.5% |
|  |  | N | 129 | 157 | 176 |  |  |  | 199 | 188 | 849 |
|  |  | % in ESS round | 100.0% | 100.0% | 100.0% |  |  |  | 100.0% | 100.0% | 100.0% |
| Belgium | Allow many to come and live here | N | 14 | 22 | 24 | 31 | 22 | 19 | 14 | 43 | 189 |
|  |  | % in ESS round | 7.4% | 9.4% | 11.6% | 14.2% | 10.3% | 8.7% | 6.7% | 20.7% | 11.1% |
|  | Allow some | N | 95 | 100 | 95 | 107 | 93 | 106 | 99 | 109 | 804 |
|  |  | % in ESS round | 50.5% | 42.6% | 45.9% | 48.9% | 43.7% | 48.4% | 47.4% | 52.4% | 47.3% |
|  | Allow a few | N | 61 | 78 | 60 | 58 | 70 | 65 | 64 | 44 | 500 |
|  |  | % in ESS round | 32.4% | 33.2% | 29.0% | 26.5% | 32.9% | 29.7% | 30.6% | 21.2% | 29.4% |
|  | Allow none | N | 18 | 35 | 28 | 23 | 28 | 29 | 32 | 12 | 205 |
|  |  | % in ESS round | 9.6% | 14.9% | 13.5% | 10.5% | 13.1% | 13.2% | 15.3% | 5.8% | 12.1% |
|  |  | N | 188 | 235 | 207 | 219 | 213 | 219 | 209 | 208 | 1698 |
|  |  | % in ESS round | 100.0% | 100.0% | 100.0% | 100.0% | 100.0% | 100.0% | 100.0% | 100.0% | 100.0% |
| Bulgaria | Allow many to come and live here | N |  |  | 25 | 29 | 31 | 28 |  |  | 113 |
|  |  | % in ESS round |  |  | 29.4% | 29.9% | 27.2% | 24.6% |  |  | 27.6% |
|  | Allow some | N |  |  | 26 | 30 | 34 | 34 |  |  | 124 |
|  |  | % in ESS round |  |  | 30.6% | 30.9% | 29.8% | 29.8% |  |  | 30.2% |
|  | Allow a few | N |  |  | 16 | 19 | 30 | 27 |  |  | 92 |
|  |  | % in ESS round |  |  | 18.8% | 19.6% | 26.3% | 23.7% |  |  | 22.4% |
|  | Allow none | N |  |  | 18 | 19 | 19 | 25 |  |  | 81 |
|  |  | % in ESS round |  |  | 21.2% | 19.6% | 16.7% | 21.9% |  |  | 19.8% |
|  |  | N |  |  | 85 | 97 | 114 | 114 |  |  | 410 |
|  |  | % in ESS round |  |  | 100.0% | 100.0% | 100.0% | 100.0% |  |  | 100.0% |
| Switzerland | Allow many to come and live here | N | 15 | 15 | 16 | 13 | 18 | 13 | 14 | 20 | 124 |
|  |  | % in ESS round | 11.9% | 10.3% | 12.0% | 9.6% | 13.6% | 9.0% | 10.8% | 15.6% | 11.5% |
|  | Allow some | N | 71 | 75 | 64 | 69 | 62 | 73 | 60 | 61 | 535 |
|  |  | % in ESS round | 56.3% | 51.4% | 48.1% | 50.7% | 47.0% | 50.3% | 46.2% | 47.7% | 49.7% |
|  | Allow a few | N | 38 | 48 | 45 | 44 | 42 | 50 | 48 | 37 | 352 |
|  |  | % in ESS round | 30.2% | 32.9% | 33.8% | 32.4% | 31.8% | 34.5% | 36.9% | 28.9% | 32.7% |
|  | Allow none | N | 2 | 8 | 8 | 10 | 10 | 9 | 8 | 10 | 65 |
|  |  | % in ESS round | 1.6% | 5.5% | 6.0% | 7.4% | 7.6% | 6.2% | 6.2% | 7.8% | 6.0% |
|  |  | N | 126 | 146 | 133 | 136 | 132 | 145 | 130 | 128 | 1076 |
|  |  | % in ESS round | 100.0% | 100.0% | 100.0% | 100.0% | 100.0% | 100.0% | 100.0% | 100.0% | 100.0% |
| Cyprus | Allow many to come and live here | N |  |  | 0 | 0 | 0 | 0 |  |  | 0 |
|  |  | % in ESS round |  |  | 0.0% | 0.0% | 0.0% | 0.0% |  |  | 0.0% |
|  | Allow some | N |  |  | 1 | 2 | 1 | 1 |  |  | 5 |
|  |  | % in ESS round |  |  | 6.3% | 11.1% | 7.7% | 7.7% |  |  | 8.3% |
|  | Allow a few | N |  |  | 10 | 10 | 7 | 6 |  |  | 33 |
|  |  | % in ESS round |  |  | 62.5% | 55.6% | 53.8% | 46.2% |  |  | 55.0% |
|  | Allow none | N |  |  | 5 | 6 | 5 | 6 |  |  | 22 |
|  |  | % in ESS round |  |  | 31.3% | 33.3% | 38.5% | 46.2% |  |  | 36.7% |
|  |  | N |  |  | 16 | 18 | 13 | 13 |  |  | 60 |
|  |  | % in ESS round |  |  | 100.0% | 100.0% | 100.0% | 100.0% |  |  | 100.0% |
| Czechia | Allow many to come and live here | N | 13 | 11 |  | 14 | 17 | 9 | 6 | 8 | 78 |
|  |  | % in ESS round | 7.9% | 6.9% |  | 5.9% | 6.7% | 5.0% | 2.5% | 2.4% | 5.0% |
|  | Allow some | N | 77 | 56 |  | 75 | 75 | 46 | 74 | 72 | 475 |
|  |  | % in ESS round | 46.7% | 35.0% |  | 31.6% | 29.4% | 25.4% | 30.8% | 21.9% | 30.3% |
|  | Allow a few | N | 57 | 64 |  | 97 | 99 | 84 | 98 | 155 | 654 |
|  |  | % in ESS round | 34.5% | 40.0% |  | 40.9% | 38.8% | 46.4% | 40.8% | 47.1% | 41.7% |
|  | Allow none | N | 18 | 29 |  | 51 | 64 | 42 | 62 | 94 | 360 |
|  |  | % in ESS round | 10.9% | 18.1% |  | 21.5% | 25.1% | 23.2% | 25.8% | 28.6% | 23.0% |
|  |  | N | 165 | 160 |  | 237 | 255 | 181 | 240 | 329 | 1567 |
|  |  | % in ESS round | 100.0% | 100.0% |  | 100.0% | 100.0% | 100.0% | 100.0% | 100.0% | 100.0% |
| Germany | Allow many to come and live here | N | 239 | 187 | 178 | 359 | 283 | 337 | 472 | 443 | 2498 |
|  |  | % in ESS round | 12.4% | 10.1% | 9.4% | 18.5% | 15.6% | 18.8% | 25.7% | 23.8% | 16.7% |
|  | Allow some | N | 955 | 763 | 743 | 837 | 843 | 870 | 837 | 849 | 6697 |
|  |  | % in ESS round | 49.4% | 41.2% | 39.3% | 43.1% | 46.3% | 48.5% | 45.6% | 45.6% | 44.9% |
|  | Allow a few | N | 619 | 655 | 678 | 563 | 536 | 489 | 440 | 492 | 4472 |
|  |  | % in ESS round | 32.0% | 35.3% | 35.8% | 29.0% | 29.5% | 27.3% | 24.0% | 26.4% | 30.0% |
|  | Allow none | N | 122 | 248 | 293 | 181 | 157 | 97 | 86 | 78 | 1262 |
|  |  | % in ESS round | 6.3% | 13.4% | 15.5% | 9.3% | 8.6% | 5.4% | 4.7% | 4.2% | 8.5% |
|  |  | N | 1935 | 1853 | 1892 | 1940 | 1819 | 1793 | 1835 | 1862 | 14929 |
|  |  | % in ESS round | 100.0% | 100.0% | 100.0% | 100.0% | 100.0% | 100.0% | 100.0% | 100.0% | 100.0% |
| Denmark | Allow many to come and live here | N | 15 | 16 | 11 | 17 | 17 | 19 | 15 |  | 110 |
|  |  | % in ESS round | 11.0% | 11.7% | 9.2% | 13.8% | 14.3% | 14.8% | 11.7% |  | 12.3% |
|  | Allow some | N | 51 | 49 | 49 | 49 | 50 | 49 | 43 |  | 340 |
|  |  | % in ESS round | 37.5% | 35.8% | 40.8% | 39.8% | 42.0% | 38.3% | 33.6% |  | 38.2% |
|  | Allow a few | N | 62 | 60 | 51 | 53 | 43 | 53 | 57 |  | 379 |
|  |  | % in ESS round | 45.6% | 43.8% | 42.5% | 43.1% | 36.1% | 41.4% | 44.5% |  | 42.5% |
|  | Allow none | N | 8 | 12 | 9 | 4 | 9 | 7 | 13 |  | 62 |
|  |  | % in ESS round | 5.9% | 8.8% | 7.5% | 3.3% | 7.6% | 5.5% | 10.2% |  | 7.0% |
|  |  | N | 136 | 137 | 120 | 123 | 119 | 128 | 128 |  | 891 |
|  |  | % in ESS round | 100.0% | 100.0% | 100.0% | 100.0% | 100.0% | 100.0% | 100.0% |  | 100.0% |
| Estonia | Allow many to come and live here | N |  | 1 | 1 | 1 | 1 | 2 | 1 | 1 | 8 |
|  |  | % in ESS round |  | 5.9% | 6.3% | 5.3% | 4.8% | 9.5% | 6.3% | 4.5% | 6.1% |
|  | Allow some | N |  | 4 | 5 | 5 | 6 | 6 | 6 | 8 | 40 |
|  |  | % in ESS round |  | 23.5% | 31.3% | 26.3% | 28.6% | 28.6% | 37.5% | 36.4% | 30.3% |
|  | Allow a few | N |  | 7 | 7 | 7 | 8 | 8 | 6 | 9 | 52 |
|  |  | % in ESS round |  | 41.2% | 43.8% | 36.8% | 38.1% | 38.1% | 37.5% | 40.9% | 39.4% |
|  | Allow none | N |  | 5 | 3 | 6 | 6 | 5 | 3 | 4 | 32 |
|  |  | % in ESS round |  | 29.4% | 18.8% | 31.6% | 28.6% | 23.8% | 18.8% | 18.2% | 24.2% |
|  |  | N |  | 17 | 16 | 19 | 21 | 21 | 16 | 22 | 132 |
|  |  | % in ESS round |  | 100.0% | 100.0% | 100.0% | 100.0% | 100.0% | 100.0% | 100.0% | 100.0% |
| Spain | Allow many to come and live here | N | 130 | 187 | 160 | 104 | 211 | 261 | 250 | 295 | 1598 |
|  |  | % in ESS round | 16.9% | 20.4% | 16.5% | 11.6% | 19.3% | 27.1% | 26.9% | 36.5% | 21.8% |
|  | Allow some | N | 255 | 328 | 387 | 331 | 387 | 329 | 338 | 271 | 2626 |
|  |  | % in ESS round | 33.2% | 35.8% | 39.9% | 36.8% | 35.4% | 34.2% | 36.4% | 33.5% | 35.8% |
|  | Allow a few | N | 330 | 327 | 307 | 361 | 380 | 278 | 242 | 200 | 2425 |
|  |  | % in ESS round | 43.0% | 35.7% | 31.6% | 40.2% | 34.8% | 28.9% | 26.1% | 24.8% | 33.0% |
|  | Allow none | N | 53 | 74 | 117 | 103 | 115 | 94 | 98 | 42 | 696 |
|  |  | % in ESS round | 6.9% | 8.1% | 12.0% | 11.5% | 10.5% | 9.8% | 10.6% | 5.2% | 9.5% |
|  |  | N | 768 | 916 | 971 | 899 | 1093 | 962 | 928 | 808 | 7345 |
|  |  | % in ESS round | 100.0% | 100.0% | 100.0% | 100.0% | 100.0% | 100.0% | 100.0% | 100.0% | 100.0% |
| Finland | Allow many to come and live here | N | 12 | 13 | 10 | 13 | 6 | 12 | 14 | 17 | 97 |
|  |  | % in ESS round | 8.2% | 9.4% | 6.5% | 9.0% | 4.5% | 8.8% | 11.3% | 12.7% | 8.8% |
|  | Allow some | N | 49 | 41 | 48 | 48 | 42 | 44 | 35 | 50 | 357 |
|  |  | % in ESS round | 33.6% | 29.7% | 31.4% | 33.3% | 31.6% | 32.4% | 28.2% | 37.3% | 32.2% |
|  | Allow a few | N | 76 | 71 | 82 | 72 | 69 | 68 | 63 | 57 | 558 |
|  |  | % in ESS round | 52.1% | 51.4% | 53.6% | 50.0% | 51.9% | 50.0% | 50.8% | 42.5% | 50.4% |
|  | Allow none | N | 9 | 13 | 13 | 11 | 16 | 12 | 12 | 10 | 96 |
|  |  | % in ESS round | 6.2% | 9.4% | 8.5% | 7.6% | 12.0% | 8.8% | 9.7% | 7.5% | 8.7% |
|  |  | N | 146 | 138 | 153 | 144 | 133 | 136 | 124 | 134 | 1108 |
|  |  | % in ESS round | 100.0% | 100.0% | 100.0% | 100.0% | 100.0% | 100.0% | 100.0% | 100.0% | 100.0% |
| France | Allow many to come and live here | N | 86 | 110 | 95 | 106 | 110 | 120 | 92 | 160 | 879 |
|  |  | % in ESS round | 8.6% | 10.6% | 9.0% | 9.9% | 10.4% | 12.4% | 12.3% | 16.0% | 11.1% |
|  | Allow some | N | 481 | 479 | 456 | 508 | 427 | 448 | 311 | 491 | 3601 |
|  |  | % in ESS round | 48.0% | 46.0% | 43.3% | 47.2% | 40.3% | 46.2% | 41.6% | 49.1% | 45.3% |
|  | Allow a few | N | 309 | 328 | 378 | 341 | 382 | 273 | 216 | 199 | 2426 |
|  |  | % in ESS round | 30.8% | 31.5% | 35.9% | 31.7% | 36.1% | 28.1% | 28.9% | 19.9% | 30.5% |
|  | Allow none | N | 126 | 124 | 124 | 121 | 140 | 129 | 128 | 150 | 1042 |
|  |  | % in ESS round | 12.6% | 11.9% | 11.8% | 11.2% | 13.2% | 13.3% | 17.1% | 15.0% | 13.1% |
|  |  | N | 1002 | 1041 | 1053 | 1076 | 1059 | 970 | 747 | 1000 | 7948 |
|  |  | % in ESS round | 100.0% | 100.0% | 100.0% | 100.0% | 100.0% | 100.0% | 100.0% | 100.0% | 100.0% |
| United Kingdom | Allow many to come and live here | N | 109 | 71 | 76 | 78 | 76 | 80 | 94 | 186 | 770 |
|  |  | % in ESS round | 9.7% | 7.8% | 7.9% | 7.1% | 8.0% | 8.9% | 10.7% | 19.0% | 9.9% |
|  | Allow some | N | 483 | 377 | 444 | 515 | 390 | 363 | 329 | 457 | 3358 |
|  |  | % in ESS round | 42.9% | 41.2% | 45.9% | 47.0% | 40.9% | 40.6% | 37.5% | 46.7% | 43.0% |
|  | Allow a few | N | 400 | 343 | 336 | 354 | 302 | 303 | 279 | 270 | 2587 |
|  |  | % in ESS round | 35.6% | 37.5% | 34.7% | 32.3% | 31.7% | 33.9% | 31.8% | 27.6% | 33.1% |
|  | Allow none | N | 133 | 124 | 112 | 149 | 186 | 149 | 175 | 66 | 1094 |
|  |  | % in ESS round | 11.8% | 13.6% | 11.6% | 13.6% | 19.5% | 16.6% | 20.0% | 6.7% | 14.0% |
|  |  | N | 1125 | 915 | 968 | 1096 | 954 | 895 | 877 | 979 | 7809 |
|  |  | % in ESS round | 100.0% | 100.0% | 100.0% | 100.0% | 100.0% | 100.0% | 100.0% | 100.0% | 100.0% |
| Greece | Allow many to come and live here | N | 5 | 9 |  | 10 | 8 |  |  |  | 32 |
|  |  | % in ESS round | 3.4% | 4.8% |  | 4.7% | 4.4% |  |  |  | 4.4% |
|  | Allow some | N | 16 | 26 |  | 24 | 21 |  |  |  | 87 |
|  |  | % in ESS round | 10.9% | 13.9% |  | 11.2% | 11.5% |  |  |  | 11.9% |
|  | Allow a few | N | 99 | 108 |  | 113 | 89 |  |  |  | 409 |
|  |  | % in ESS round | 67.3% | 57.8% |  | 52.8% | 48.9% |  |  |  | 56.0% |
|  | Allow none | N | 27 | 44 |  | 67 | 64 |  |  |  | 202 |
|  |  | % in ESS round | 18.4% | 23.5% |  | 31.3% | 35.2% |  |  |  | 27.7% |
|  |  | N | 147 | 187 |  | 214 | 182 |  |  |  | 730 |
|  |  | % in ESS round | 100.0% | 100.0% |  | 100.0% | 100.0% |  |  |  | 100.0% |
| Croatia | Allow many to come and live here | N |  |  |  | 14 | 15 |  |  |  | 29 |
|  |  | % in ESS round |  |  |  | 22.6% | 25.9% |  |  |  | 24.2% |
|  | Allow some | N |  |  |  | 26 | 19 |  |  |  | 45 |
|  |  | % in ESS round |  |  |  | 41.9% | 32.8% |  |  |  | 37.5% |
|  | Allow a few | N |  |  |  | 14 | 16 |  |  |  | 30 |
|  |  | % in ESS round |  |  |  | 22.6% | 27.6% |  |  |  | 25.0% |
|  | Allow none | N |  |  |  | 8 | 8 |  |  |  | 16 |
|  |  | % in ESS round |  |  |  | 12.9% | 13.8% |  |  |  | 13.3% |
|  |  | N |  |  |  | 62 | 58 |  |  |  | 120 |
|  |  | % in ESS round |  |  |  | 100.0% | 100.0% |  |  |  | 100.0% |
| Hungary | Allow many to come and live here | N | 2 | 7 | 5 | 8 | 11 | 6 | 6 | 3 | 48 |
|  |  | % in ESS round | 1.3% | 3.6% | 2.8% | 3.9% | 4.7% | 2.8% | 2.6% | 1.3% | 2.9% |
|  | Allow some | N | 20 | 23 | 20 | 23 | 35 | 41 | 27 | 12 | 201 |
|  |  | % in ESS round | 13.2% | 11.7% | 11.3% | 11.1% | 15.1% | 18.8% | 11.5% | 5.4% | 12.3% |
|  | Allow a few | N | 99 | 106 | 85 | 99 | 107 | 97 | 92 | 65 | 750 |
|  |  | % in ESS round | 65.1% | 54.1% | 48.0% | 47.8% | 46.1% | 44.5% | 39.3% | 29.0% | 45.7% |
|  | Allow none | N | 31 | 60 | 67 | 77 | 79 | 74 | 109 | 144 | 641 |
|  |  | % in ESS round | 20.4% | 30.6% | 37.9% | 37.2% | 34.1% | 33.9% | 46.6% | 64.3% | 39.1% |
|  |  | N | 152 | 196 | 177 | 207 | 232 | 218 | 234 | 224 | 1640 |
|  |  | % in ESS round | 100.0% | 100.0% | 100.0% | 100.0% | 100.0% | 100.0% | 100.0% | 100.0% | 100.0% |
| Ireland | Allow many to come and live here | N | 9 | 14 | 9 | 8 | 6 | 12 | 7 | 8 | 73 |
|  |  | % in ESS round | 14.5% | 20.3% | 15.8% | 11.3% | 7.3% | 15.2% | 10.8% | 11.9% | 13.2% |
|  | Allow some | N | 33 | 31 | 31 | 33 | 36 | 34 | 19 | 29 | 246 |
|  |  | % in ESS round | 53.2% | 44.9% | 54.4% | 46.5% | 43.9% | 43.0% | 29.2% | 43.3% | 44.6% |
|  | Allow a few | N | 17 | 19 | 13 | 21 | 24 | 24 | 27 | 23 | 168 |
|  |  | % in ESS round | 27.4% | 27.5% | 22.8% | 29.6% | 29.3% | 30.4% | 41.5% | 34.3% | 30.4% |
|  | Allow none | N | 3 | 5 | 4 | 9 | 16 | 9 | 12 | 7 | 65 |
|  |  | % in ESS round | 4.8% | 7.2% | 7.0% | 12.7% | 19.5% | 11.4% | 18.5% | 10.4% | 11.8% |
|  |  | N | 62 | 69 | 57 | 71 | 82 | 79 | 65 | 67 | 552 |
|  |  | % in ESS round | 100.0% | 100.0% | 100.0% | 100.0% | 100.0% | 100.0% | 100.0% | 100.0% | 100.0% |
| Israel | Allow many to come and live here | N | 3 |  |  | 2 | 2 | 2 | 4 | 5 | 18 |
|  |  | % in ESS round | 18.8% |  |  | 7.7% | 6.1% | 4.1% | 7.5% | 11.4% | 8.1% |
|  | Allow some | N | 5 |  |  | 7 | 9 | 8 | 12 | 13 | 54 |
|  |  | % in ESS round | 31.3% |  |  | 26.9% | 27.3% | 16.3% | 22.6% | 29.5% | 24.4% |
|  | Allow a few | N | 4 |  |  | 8 | 10 | 13 | 20 | 11 | 66 |
|  |  | % in ESS round | 25.0% |  |  | 30.8% | 30.3% | 26.5% | 37.7% | 25.0% | 29.9% |
|  | Allow none | N | 4 |  |  | 9 | 12 | 26 | 17 | 15 | 83 |
|  |  | % in ESS round | 25.0% |  |  | 34.6% | 36.4% | 53.1% | 32.1% | 34.1% | 37.6% |
|  |  | N | 16 |  |  | 26 | 33 | 49 | 53 | 44 | 221 |
|  |  | % in ESS round | 100.0% |  |  | 100.0% | 100.0% | 100.0% | 100.0% | 100.0% | 100.0% |
| Iceland | Allow many to come and live here | N |  | 1 |  |  |  | 2 |  | 3 | 6 |
|  |  | % in ESS round |  | 25.0% |  |  |  | 50.0% |  | 60.0% | 46.2% |
|  | Allow some | N |  | 2 |  |  |  | 1 |  | 2 | 5 |
|  |  | % in ESS round |  | 50.0% |  |  |  | 25.0% |  | 40.0% | 38.5% |
|  | Allow a few | N |  | 1 |  |  |  | 1 |  | 0 | 2 |
|  |  | % in ESS round |  | 25.0% |  |  |  | 25.0% |  | 0.0% | 15.4% |
|  | Allow none | N |  | 0 |  |  |  | 0 |  | 0 | 0 |
|  |  | % in ESS round |  | 0.0% |  |  |  | 0.0% |  | 0.0% | 0.0% |
|  |  | N |  | 4 |  |  |  | 4 |  | 5 | 13 |
|  |  | % in ESS round |  | 100.0% |  |  |  | 100.0% |  | 100.0% | 100.0% |
| Italy | Allow many to come and live here | N | 259 |  |  |  |  | 245 |  | 138 | 642 |
|  |  | % in ESS round | 16.8% |  |  |  |  | 20.4% |  | 12.5% | 16.7% |
|  | Allow some | N | 684 |  |  |  |  | 567 |  | 386 | 1637 |
|  |  | % in ESS round | 44.3% |  |  |  |  | 47.3% |  | 35.0% | 42.6% |
|  | Allow a few | N | 488 |  |  |  |  | 229 |  | 377 | 1094 |
|  |  | % in ESS round | 31.6% |  |  |  |  | 19.1% |  | 34.2% | 28.4% |
|  | Allow none | N | 114 |  |  |  |  | 158 |  | 202 | 474 |
|  |  | % in ESS round | 7.4% |  |  |  |  | 13.2% |  | 18.3% | 12.3% |
|  |  | N | 1545 |  |  |  |  | 1199 |  | 1103 | 3847 |
|  |  | % in ESS round | 100.0% |  |  |  |  | 100.0% |  | 100.0% | 100.0% |
| Lithuania | Allow many to come and live here | N |  |  |  |  | 9 | 5 | 4 | 3 | 21 |
|  |  | % in ESS round |  |  |  |  | 17.3% | 12.8% | 9.3% | 7.1% | 11.9% |
|  | Allow some | N |  |  |  |  | 21 | 17 | 13 | 12 | 63 |
|  |  | % in ESS round |  |  |  |  | 40.4% | 43.6% | 30.2% | 28.6% | 35.8% |
|  | Allow a few | N |  |  |  |  | 13 | 10 | 14 | 16 | 53 |
|  |  | % in ESS round |  |  |  |  | 25.0% | 25.6% | 32.6% | 38.1% | 30.1% |
|  | Allow none | N |  |  |  |  | 9 | 7 | 12 | 11 | 39 |
|  |  | % in ESS round |  |  |  |  | 17.3% | 17.9% | 27.9% | 26.2% | 22.2% |
|  |  | N |  |  |  |  | 52 | 39 | 43 | 42 | 176 |
|  |  | % in ESS round |  |  |  |  | 100.0% | 100.0% | 100.0% | 100.0% | 100.0% |
| Luxembourg | Allow many to come and live here | N | 1 | 1 |  |  |  |  |  |  | 2 |
|  |  | % in ESS round | 33.3% | 20.0% |  |  |  |  |  |  | 25.0% |
|  | Allow some | N | 1 | 2 |  |  |  |  |  |  | 3 |
|  |  | % in ESS round | 33.3% | 40.0% |  |  |  |  |  |  | 37.5% |
|  | Allow a few | N | 1 | 1 |  |  |  |  |  |  | 2 |
|  |  | % in ESS round | 33.3% | 20.0% |  |  |  |  |  |  | 25.0% |
|  | Allow none | N | 0 | 1 |  |  |  |  |  |  | 1 |
|  |  | % in ESS round | 0.0% | 20.0% |  |  |  |  |  |  | 12.5% |
|  |  | N | 3 | 5 |  |  |  |  |  |  | 8 |
|  |  | % in ESS round | 100.0% | 100.0% |  |  |  |  |  |  | 100.0% |
| Netherlands | Allow many to come and live here | N | 23 | 25 | 32 | 32 | 31 | 41 | 26 | 50 | 260 |
|  |  | % in ESS round | 6.7% | 7.1% | 8.7% | 10.0% | 10.2% | 12.4% | 9.5% | 16.2% | 10.0% |
|  | Allow some | N | 181 | 162 | 153 | 153 | 145 | 146 | 116 | 138 | 1194 |
|  |  | % in ESS round | 52.6% | 46.2% | 41.6% | 47.7% | 47.9% | 44.2% | 42.5% | 44.7% | 45.9% |
|  | Allow a few | N | 114 | 121 | 139 | 113 | 89 | 108 | 96 | 94 | 874 |
|  |  | % in ESS round | 33.1% | 34.5% | 37.8% | 35.2% | 29.4% | 32.7% | 35.2% | 30.4% | 33.6% |
|  | Allow none | N | 26 | 43 | 44 | 23 | 38 | 35 | 35 | 27 | 271 |
|  |  | % in ESS round | 7.6% | 12.3% | 12.0% | 7.2% | 12.5% | 10.6% | 12.8% | 8.7% | 10.4% |
|  |  | N | 344 | 351 | 368 | 321 | 303 | 330 | 273 | 309 | 2599 |
|  |  | % in ESS round | 100.0% | 100.0% | 100.0% | 100.0% | 100.0% | 100.0% | 100.0% | 100.0% | 100.0% |
| Norway | Allow many to come and live here | N | 15 | 12 | 17 | 20 | 15 | 19 | 19 | 29 | 146 |
|  |  | % in ESS round | 13.3% | 10.5% | 15.3% | 17.1% | 14.2% | 18.3% | 17.3% | 26.9% | 16.5% |
|  | Allow some | N | 57 | 53 | 53 | 56 | 54 | 52 | 59 | 54 | 438 |
|  |  | % in ESS round | 50.4% | 46.5% | 47.7% | 47.9% | 50.9% | 50.0% | 53.6% | 50.0% | 49.6% |
|  | Allow a few | N | 38 | 40 | 38 | 37 | 32 | 29 | 30 | 23 | 267 |
|  |  | % in ESS round | 33.6% | 35.1% | 34.2% | 31.6% | 30.2% | 27.9% | 27.3% | 21.3% | 30.2% |
|  | Allow none | N | 3 | 9 | 3 | 4 | 5 | 4 | 2 | 2 | 32 |
|  |  | % in ESS round | 2.7% | 7.9% | 2.7% | 3.4% | 4.7% | 3.8% | 1.8% | 1.9% | 3.6% |
|  |  | N | 113 | 114 | 111 | 117 | 106 | 104 | 110 | 108 | 883 |
|  |  | % in ESS round | 100.0% | 100.0% | 100.0% | 100.0% | 100.0% | 100.0% | 100.0% | 100.0% | 100.0% |
| Poland | Allow many to come and live here | N | 76 | 133 | 211 | 210 | 184 | 194 | 107 | 62 | 1177 |
|  |  | % in ESS round | 11.2% | 19.1% | 28.9% | 25.7% | 23.4% | 25.8% | 13.0% | 8.3% | 19.5% |
|  | Allow some | N | 357 | 360 | 355 | 430 | 440 | 362 | 340 | 345 | 2989 |
|  |  | % in ESS round | 52.4% | 51.6% | 48.6% | 52.6% | 55.8% | 48.2% | 41.3% | 46.0% | 49.5% |
|  | Allow a few | N | 200 | 171 | 139 | 147 | 146 | 149 | 284 | 278 | 1514 |
|  |  | % in ESS round | 29.4% | 24.5% | 19.0% | 18.0% | 18.5% | 19.8% | 34.5% | 37.1% | 25.1% |
|  | Allow none | N | 48 | 33 | 26 | 30 | 18 | 46 | 93 | 65 | 359 |
|  |  | % in ESS round | 7.0% | 4.7% | 3.6% | 3.7% | 2.3% | 6.1% | 11.3% | 8.7% | 5.9% |
|  |  | N | 681 | 697 | 731 | 817 | 788 | 751 | 824 | 750 | 6039 |
|  |  | % in ESS round | 100.0% | 100.0% | 100.0% | 100.0% | 100.0% | 100.0% | 100.0% | 100.0% | 100.0% |
| Portugal | Allow many to come and live here | N | 9 | 3 | 11 | 13 | 8 | 8 | 16 | 32 | 100 |
|  |  | % in ESS round | 3.9% | 2.1% | 5.3% | 9.2% | 5.5% | 4.7% | 12.4% | 11.8% | 7.0% |
|  | Allow some | N | 72 | 45 | 64 | 47 | 54 | 47 | 46 | 162 | 537 |
|  |  | % in ESS round | 31.4% | 31.7% | 30.6% | 33.3% | 37.0% | 27.8% | 35.7% | 59.8% | 37.4% |
|  | Allow a few | N | 100 | 63 | 73 | 55 | 51 | 55 | 46 | 58 | 501 |
|  |  | % in ESS round | 43.7% | 44.4% | 34.9% | 39.0% | 34.9% | 32.5% | 35.7% | 21.4% | 34.9% |
|  | Allow none | N | 48 | 31 | 61 | 26 | 33 | 59 | 21 | 19 | 298 |
|  |  | % in ESS round | 21.0% | 21.8% | 29.2% | 18.4% | 22.6% | 34.9% | 16.3% | 7.0% | 20.8% |
|  |  | N | 229 | 142 | 209 | 141 | 146 | 169 | 129 | 271 | 1436 |
|  |  | % in ESS round | 100.0% | 100.0% | 100.0% | 100.0% | 100.0% | 100.0% | 100.0% | 100.0% | 100.0% |
| Russia | Allow many to come and live here | N |  |  | 150 | 197 | 249 | 252 |  | 142 | 990 |
|  |  | % in ESS round |  |  | 9.1% | 10.8% | 11.6% | 9.4% |  | 5.8% | 9.2% |
|  | Allow some | N |  |  | 346 | 444 | 531 | 627 |  | 570 | 2518 |
|  |  | % in ESS round |  |  | 21.0% | 24.4% | 24.7% | 23.3% |  | 23.3% | 23.4% |
|  | Allow a few | N |  |  | 568 | 613 | 793 | 907 |  | 977 | 3858 |
|  |  | % in ESS round |  |  | 34.5% | 33.7% | 36.8% | 33.7% |  | 40.0% | 35.9% |
|  | Allow none | N |  |  | 583 | 565 | 580 | 909 |  | 755 | 3392 |
|  |  | % in ESS round |  |  | 35.4% | 31.1% | 26.9% | 33.7% |  | 30.9% | 31.5% |
|  |  | N |  |  | 1647 | 1819 | 2153 | 2695 |  | 2444 | 10758 |
|  |  | % in ESS round |  |  | 100.0% | 100.0% | 100.0% | 100.0% |  | 100.0% | 100.0% |
| Sweden | Allow many to come and live here | N | 61 | 64 | 68 | 58 | 64 | 59 | 74 | 53 | 501 |
|  |  | % in ESS round | 31.0% | 31.4% | 36.6% | 33.9% | 35.6% | 35.3% | 41.1% | 34.0% | 34.8% |
|  | Allow some | N | 114 | 108 | 89 | 93 | 100 | 84 | 89 | 87 | 764 |
|  |  | % in ESS round | 57.9% | 52.9% | 47.8% | 54.4% | 55.6% | 50.3% | 49.4% | 55.8% | 53.0% |
|  | Allow a few | N | 19 | 27 | 24 | 19 | 14 | 22 | 15 | 15 | 155 |
|  |  | % in ESS round | 9.6% | 13.2% | 12.9% | 11.1% | 7.8% | 13.2% | 8.3% | 9.6% | 10.8% |
|  | Allow none | N | 3 | 5 | 5 | 1 | 2 | 2 | 2 | 1 | 21 |
|  |  | % in ESS round | 1.5% | 2.5% | 2.7% | .6% | 1.1% | 1.2% | 1.1% | .6% | 1.5% |
|  |  | N | 197 | 204 | 186 | 171 | 180 | 167 | 180 | 156 | 1441 |
|  |  | % in ESS round | 100.0% | 100.0% | 100.0% | 100.0% | 100.0% | 100.0% | 100.0% | 100.0% | 100.0% |
| Slovenia | Allow many to come and live here | N | 3 |  | 2 | 4 | 3 | 4 | 5 | 4 | 25 |
|  |  | % in ESS round | 11.5% |  | 10.0% | 13.3% | 11.5% | 12.9% | 14.7% | 12.5% | 12.6% |
|  | Allow some | N | 13 |  | 11 | 14 | 13 | 14 | 16 | 15 | 96 |
|  |  | % in ESS round | 50.0% |  | 55.0% | 46.7% | 50.0% | 45.2% | 47.1% | 46.9% | 48.2% |
|  | Allow a few | N | 8 |  | 6 | 9 | 8 | 10 | 9 | 10 | 60 |
|  |  | % in ESS round | 30.8% |  | 30.0% | 30.0% | 30.8% | 32.3% | 26.5% | 31.3% | 30.2% |
|  | Allow none | N | 2 |  | 1 | 3 | 2 | 3 | 4 | 3 | 18 |
|  |  | % in ESS round | 7.7% |  | 5.0% | 10.0% | 7.7% | 9.7% | 11.8% | 9.4% | 9.0% |
|  |  | N | 26 |  | 20 | 30 | 26 | 31 | 34 | 32 | 199 |
|  |  | % in ESS round | 100.0% |  | 100.0% | 100.0% | 100.0% | 100.0% | 100.0% | 100.0% | 100.0% |
| Slovakia | Allow many to come and live here | N |  | 15 | 23 | 23 | 13 | 12 |  |  | 86 |
|  |  | % in ESS round |  | 20.3% | 20.7% | 20.7% | 13.0% | 9.8% |  |  | 16.6% |
|  | Allow some | N |  | 35 | 48 | 40 | 36 | 33 |  |  | 192 |
|  |  | % in ESS round |  | 47.3% | 43.2% | 36.0% | 36.0% | 26.8% |  |  | 37.0% |
|  | Allow a few | N |  | 19 | 27 | 35 | 37 | 50 |  |  | 168 |
|  |  | % in ESS round |  | 25.7% | 24.3% | 31.5% | 37.0% | 40.7% |  |  | 32.4% |
|  | Allow none | N |  | 5 | 13 | 13 | 14 | 28 |  |  | 73 |
|  |  | % in ESS round |  | 6.8% | 11.7% | 11.7% | 14.0% | 22.8% |  |  | 14.1% |
|  |  | N |  | 74 | 111 | 111 | 100 | 123 |  |  | 519 |
|  |  | % in ESS round |  | 100.0% | 100.0% | 100.0% | 100.0% | 100.0% |  |  | 100.0% |
| Turkey | Allow many to come and live here | N |  | 122 |  | 136 |  |  |  |  | 258 |
|  |  | % in ESS round |  | 11.7% |  | 10.4% |  |  |  |  | 11.0% |
|  | Allow some | N |  | 237 |  | 355 |  |  |  |  | 592 |
|  |  | % in ESS round |  | 22.7% |  | 27.2% |  |  |  |  | 25.2% |
|  | Allow a few | N |  | 185 |  | 341 |  |  |  |  | 526 |
|  |  | % in ESS round |  | 17.7% |  | 26.1% |  |  |  |  | 22.4% |
|  | Allow none | N |  | 502 |  | 473 |  |  |  |  | 975 |
|  |  | % in ESS round |  | 48.0% |  | 36.2% |  |  |  |  | 41.5% |
|  |  | N |  | 1046 |  | 1305 |  |  |  |  | 2351 |
|  |  | % in ESS round |  | 100.0% |  | 100.0% |  |  |  |  | 100.0% |
| Ukraine | Allow many to come and live here | N |  | 126 | 134 | 65 | 49 | 58 |  |  | 432 |
|  |  | % in ESS round |  | 23.7% | 30.6% | 15.6% | 13.2% | 13.2% |  |  | 19.7% |
|  | Allow some | N |  | 170 | 89 | 102 | 121 | 123 |  |  | 605 |
|  |  | % in ESS round |  | 32.0% | 20.3% | 24.5% | 32.5% | 28.1% |  |  | 27.6% |
|  | Allow a few | N |  | 128 | 115 | 152 | 99 | 128 |  |  | 622 |
|  |  | % in ESS round |  | 24.1% | 26.3% | 36.5% | 26.6% | 29.2% |  |  | 28.3% |
|  | Allow none | N |  | 108 | 100 | 97 | 103 | 129 |  |  | 537 |
|  |  | % in ESS round |  | 20.3% | 22.8% | 23.3% | 27.7% | 29.5% |  |  | 24.5% |
|  |  | N |  | 532 | 438 | 416 | 372 | 438 |  |  | 2196 |
|  |  | % in ESS round |  | 100.0% | 100.0% | 100.0% | 100.0% | 100.0% |  |  | 100.0% |
| Total | Allow many to come and live here | N | 1111 | 1187 | 1278 | 1565 | 1459 | 1819 | 1265 | 1726 | 11410 |
|  |  | % in ESS round | 12.0% | 12.7% | 13.0% | 13.2% | 13.6% | 15.2% | 17.1% | 15.4% | 14.0% |
|  | Allow some | N | 4115 | 3590 | 3643 | 4423 | 4045 | 4525 | 2931 | 4243 | 31515 |
|  |  | % in ESS round | 44.6% | 38.5% | 37.0% | 37.4% | 37.7% | 37.8% | 39.7% | 37.8% | 38.6% |
|  | Allow a few | N | 3202 | 3026 | 3267 | 3755 | 3496 | 3536 | 2215 | 3482 | 25979 |
|  |  | % in ESS round | 34.7% | 32.4% | 33.2% | 31.7% | 32.6% | 29.5% | 30.0% | 31.1% | 31.9% |
|  | Allow none | N | 807 | 1533 | 1657 | 2089 | 1738 | 2093 | 967 | 1762 | 12646 |
|  |  | % in ESS round | 8.7% | 16.4% | 16.8% | 17.7% | 16.2% | 17.5% | 13.1% | 15.7% | 15.5% |
|  |  | N | 9235 | 9336 | 9845 | 11832 | 10738 | 11973 | 7378 | 11213 | 81550 |
|  |  | % in ESS round | 100.0% | 100.0% | 100.0% | 100.0% | 100.0% | 100.0% | 100.0% | 100.0% | 100.0% |
